# Hippocampal Neurogenesis Reduces the Dimensionality of Sparsely Coded Representations to Enhance Memory Encoding

**DOI:** 10.3389/fncom.2018.00099

**Published:** 2019-01-07

**Authors:** Anthony J. DeCostanzo, Chi Chung Alan Fung, Tomoki Fukai

**Affiliations:** ^1^Laboratory for Neural Coding and Brain Computing, RIKEN Center for Brain Science, Saitama, Japan; ^2^Ascent Robotics Inc., Tokyo, Japan

**Keywords:** dimensionality reduction, hippocampus, pattern separation, neuromorphic computing, feed-forward neural network, synaptic pruning, synaptic turnover, synaptic plasticity

## Abstract

Adult neurogenesis in the hippocampal dentate gyrus (DG) of mammals is known to contribute to memory encoding in many tasks. The DG also exhibits exceptionally sparse activity compared to other systems, however, whether sparseness and neurogenesis interact during memory encoding remains elusive. We implement a novel learning rule consistent with experimental findings of competition among adult-born neurons in a supervised multilayer feedforward network trained to discriminate between contexts. From this rule, the DG population partitions into neuronal ensembles each of which is biased to represent one of the contexts. This corresponds to a low dimensional representation of the contexts, whereby the fastest dimensionality reduction is achieved in sparse models. We then modify the rule, showing that equivalent representations and performance are achieved when neurons compete for synaptic stability rather than neuronal survival. Our results suggest that competition for stability in sparse models is well-suited to developing ensembles of what may be called memory engram cells.

## 1. Introduction

### 1.1. What Is Known

The hippocampal dentate gyrus (DG) is known to participate in the generation and maintenance of spatio-contextual memories via groups of cells whose activity is causally responsible for the recollection of particular associations (Josselyn et al., 2015; Tonegawa et al., 2015). The DG is noted for a combination of distinctive properties, including adult neurogenesis of the principle granule cells (Wu et al., 2015; Gonçalves et al., 2016) and extremely sparse activity (Jung and McNaughton, 1993; Leutgeb et al., 2007; Danielson et al., 2016; Diamantaki et al., 2016).

Since most adult-born neurons rapidly die, it has long been hypothesized that they must compete amongst themselves, and with mature neurons, for survival dependent upon their contribution to behavior (Bergami and Berninger, 2012). Consistent with this notion, newly adult-born cells integrate into the DG in an experience-dependent manner (Kempermann et al., 1997b; Gould et al., 1999; Bergami et al., 2015; Alvarez et al., 2016; Zhuo et al., 2016), and numerous studies have demonstrated that either ablation (Clelland et al., 2009; Sahay et al., 2011), or *in vivo* silencing of activity (Danielson et al., 2016; Zhuo et al., 2016) or synaptic output (Nakashiba et al., 2012) of these cells impairs discrimination of hippocampus-dependent associative memories, while enhancing survival of these cells can enhance such performance (Sahay et al., 2011). Similar interventions that silence adult-born cells after learning have shown that retrieval of recent memories is impaired (Gu et al., 2012).

Experience induces synaptic competition among adult-generated granule cells for contacts to CA3 neurons resulting in axonal retraction by mature cells induced by young cells (Yasuda et al., 2011). Elsewhere in both the central (Fitzsimonds et al., 1997; Tao et al., 2000; Du and Poo, 2004) and peripheral nervous systems (Sharma et al., 2010; Zhou et al., 2012), the strength of a neurons output synapses can retrogradely adjust the strength of its input synapses. It has been suggested that this biological phenomenon could encode a neurons performance errors to achieve a similar effect to the artificial backpropagation of error so commonly employed in training neural networks (Harris, 2008). Adult-born DG granule cells reach their targets in CA3 after about 4–6 weeks (Toni et al., 2008), overlapping with when they begin to participate in memory encoding (Clelland et al., 2009; Sahay et al., 2011; Nakashiba et al., 2012; Danielson et al., 2016; Zhuo et al., 2016), and thus may begin to receive signals from CA3 that indicate the success of their contribution to useful representations. The combination of these results suggests that neurogenesis may endow the DG with a kind of learning rule—DG neurons compete with each other for target-derived factors through their synaptic contact to CA3, in turn, influencing their probability of survival. Such a learning rule is the focus of our study.

In an apparently distinct thread of research, sparse activity in recurrent Hopfield-like networks is shown to reduce the interference between stored memories (Tsodyks and Feigel'man, 1988; Amit and Fusi, 1994) and, in models of vision, to enable the efficient representation of naturalistic images as combinations of statistically independent components (Olshausen and Field, 1996; Bell and Sejnowski, 1997), ideas that have roots in the efficient coding hypothesis (Barlow, 1961). In cortical models consisting of a single hidden layer multilayer perceptron with random input weights, it has been shown that pattern decorrelation (often called pattern separation in the neurogenesis literature) is not sufficient to yield proper memory retrieval in the presence of noise (Barak et al., 2013; Babadi and Sompolinsky, 2014). Instead, memory retrieval depends upon a balance between decorrelation of input patterns and generalization of those patterns to the correct class. In such models, sparseness improves memory retrieval by reducing the tradeoff between decorrelation and generalization (Barak et al., 2013). This apparent tradeoff has been analytically expressed in terms that reflect the counterintuitive amplification of noise by sparse coding (Babadi and Sompolinsky, 2014). As a result, there is a theoretical limit on the benefits provided by sparseness in a hidden layer with random input weights (Barak et al., 2013; Babadi and Sompolinsky, 2014). This limitation led some authors to suggest that random weighting is at least partly responsible for limiting the benefits of sparse coding (Babadi and Sompolinsky, 2014).

### 1.2. Our Contribution

One interpretation of these studies is that pattern classification performance, rather than pattern separation, as it has been defined in the neurogenesis literature, may be the appropriate measure of memory performance. We hone our questions into a framework similar to that employed in previous studies of sparse cortical representations (Barak et al., 2013; Babadi and Sompolinsky, 2014), a single-hidden layer, randomly connected feedforward neural network. Within this framework we represent the activities of the neurogenic cells of the dentate gyrus in the hidden layer. With only minimal assumptions, such a network can learn generalizable, nonlinear classifications (Barak et al., 2013), while allowing us to implement sparse coding, synaptic plasticity, and competition among DG neurons for contact with CA3. By supervising the output, the network is trained and then tested for discrimination between sets of input patterns.

We first demonstrate that our neuronal turnover rule, employing randomly drawn input weights, markedly increases the discrimination performance over the initial condition of random projection that was previously studied (Barak et al., 2013; Babadi and Sompolinsky, 2014). The rule exploits sparse coding such that the longer neuronal turnover is allowed to proceed, the sparser the optimal coding level. Since our input weights are always drawn randomly, our results suggest that the sparsening of the optimal code is due to the achievement of a particular hidden layer representation rather than a structuring of the input weights, as was the case explored by Babadi and Sompolinsky. Thus our work complements theirs by suggesting a learning rule via which very sparse codes are optimal for random input weights without require fine tuning.

We show that our rule induces a contextual preference among DG neurons, partitioning the population into ensembles whose average activities are biased for their respective contexts. This is equivalent to dimensionality reduction of the contextual representations in the DG. The final classification performed by the CA3 readout thereby suffers less errors during generalization. We demonstrate that the final achievable discrimination between contextual memories is constrained by the distribution of singular values of the DG representation, such that the sparse code can evolve to a greater difference in the representation space. We then construct a more general model based on evidence that the strength of a neurons output synapses can influence that of its input synapses via internal signals (Fitzsimonds et al., 1997; Tao et al., 2000; Du and Poo, 2004; Sharma et al., 2010; Zhou et al., 2012). This rule similarly reduces the dimensionality of the representation while shifting the activity-dependence toward sparser levels, improving memory performance. Our results suggest that axonal competition for target-mediated stability in sparse models is a novel form of encoding that does not require synaptic fine-tuning, and could be employed across many sparsely coded systems of the brain.

## 2. Materials and Methods

### 2.1. Representations of Contexts

We represent the activity state of a population of EC neurons in response to a stimulus as a vector ξ, the elements of which neurons that are either spiking, ξ_*j*_ = +1, or not spiking ξ_*j*_ = −1. Patterns are split evenly into two contexts representing the two contexts that the network must learn. The synaptic current of a given DG unit *i* for pattern μ is defined as:

(1) $$\begin{array}{l} g_{i}^{\mu }=\overset{M}{\underset{j}{\sum }}J_{j}\xi_{j}^{\mu } \end{array}$$

and its activity is given by a threshold function of the synaptic current controlled by θ:

(2) $$\begin{array}{l} S_{i}^{\mu }=\text{sgn}\left(g_{i}^{\mu }-\theta \right) \end{array}$$

The CA3 synaptic current is defined similarly as the weighted sum of the input from DG:

(3) $$\begin{array}{l} h^{\mu }=\overset{N}{\underset{i}{\sum }}W_{i}S_{i}^{\mu } \end{array}$$

For every μ'th pattern we want the output of the trained network, ${\hat{\eta }}^{\mu }=\text{sgn}\left(h^{\mu }\right)$ to be equal to a randomly pre-chosen target output state for the CA3 unit, either spiking η^μ^ = +1, or not spiking, η^μ^ = −1, for all patterns.

### 2.2. Training the Network With Neurogenesis

The task of the network is to use the training patterns to find a *W* such that when presented with patterns of a given class to which the network has not been explicitly trained it can correctly generalize, i.e., it will still output the correct class. We train the CA3 output weights in a similar manner to Barak et al. (2013). We assume that the activity of the EC consists of random, uncorrelated prototype patterns, ξ, that determine their corresponding current in the DG, $g_{i}=\overset{M}{\underset{j}{\sum }}J_{j}\xi_{j}^{\mu }$. We then assume there is noise, or variability in the system such that each prototype pattern is actually represented by a group of noisy instances of the prototype that are generated by flipping the sign of elements of the vector ξ with a fixed probability ν = 0.2. This allows us to calculate the mean synaptic current of a given DG unit *i* for pattern μ as:

(4) $$\begin{array}{l} {\bar{g}}_{i}^{\mu }=g_{i}^{\mu }\left(1-2\nu \right). \end{array}$$

Consider the difference between two noisy instances of a prototype pattern, say $g_{i}^{\mu }\left(t\right)$ at the *t*-th iteration and $g_{i}^{\mu }\left(t^{\prime }\right)$ at the *t*′-th iteration:

$$\begin{array}{l} g_{i}^{\mu }\left(t\right)-g_{i}^{\mu }\left(t^{\prime }\right)\;=\underset{+1\text{ entries flipped in }t\text{ but not in }t^{\prime }}{\sum }J_{ij}\times \left(+2\right)+\underset{+1\text{ entries not flipped in }t\text{ but in }t^{\prime }}{\sum }J_{ij}\times \left(-2\right)\\ \;+\underset{-1\text{ entries flipped in }t\text{ but not in }t^{\prime }}{\sum }J_{ij}\times \left(-2\right)+\underset{-1\text{ entries not flipped in }t\text{ but in }t^{\prime }}{\sum }J_{ij}\times \left(+2\right) \end{array}$$

Here the sign accompanying “2” will be absorbed into *J*_*ij*_ to simplify the calculation because $J_{ij}\sim N\left(0,1\right)$.

$$\begin{array}{l} \langle {\left[g_{i}^{\mu }(t)-g_{i}^{\mu }\left(t^{\prime }\right)\right]}^{2}\rangle =\langle (\sum_{\text{ entries flipped in }t\text{ but not in }t^{\prime }}J_{ij}\times 2\\ {\;\;+\sum_{\text{ entries not flipped in }t\text{ but in }t^{7}{\;}^{\prime }}J_{\text{ ij }}\times 2)}^{2}\rangle \\ \;=4\langle \sum_{Mv\;(1-v)\text{ terms }}J_{ij}^{2}+\sum_{Mv\;(1-v)\text{ terms }}J_{ij}^{2}\rangle \\ \;=8Mv\text{ ( 1 - }v\text{ )}. \end{array}$$

Here $\langle J_{ij}^{2}\rangle =1$ because $J_{ij}\sim N\left(0,1\right)$. On the other hand, let $g_{i}^{\mu }\left(t\right)={\bar{g}}_{i}^{\mu }+\delta g_{i}^{\mu }\left(t\right)$, then we have

$$\begin{array}{l} \langle {\left[g_{i}^{\mu }\left(t\right)-g_{i}^{\mu }\left(t^{\prime }\right)\right]}^{2}\rangle \;=\langle \delta g_{i}^{\mu }{\left(t\right)}^{2}+\delta g_{i}^{\mu }{\left(t^{\prime }\right)}^{2}-2\delta g_{i}^{\mu }\left(t\right)\delta g_{i}^{\mu }\left(t^{\prime }\right)\rangle \\ \;=\langle \delta g_{i}^{\mu }{\left(t\right)}^{2}\rangle +\langle \delta g_{i}^{\mu }{\left(t^{\prime }\right)}^{2}\rangle \\ \;=2\langle \delta g_{i}^{\mu }{\left(t\right)}^{2}\rangle . \end{array}$$

Hence, the variance of each DG unit is given by

(5) $$\begin{array}{l} \sigma_{g}^{2}\;=\langle \delta g_{i}^{\mu }{\left(t\right)}^{2}\rangle \\ \;=4M\nu \left(1-\nu \right). \end{array}$$

Since the synaptic currents of the *i*-th DG unit for noisy instances are sum of many randomly altered numbers, those synaptic currents can be assumed to be Gaussian. The expected value of the activity of the *i*-th DG unit can be deduced by

(6) $$\begin{array}{l} {\bar{S}}_{i}^{\mu }=-1\times \int_{-\infty }^{\theta }dgf\left(g|{\bar{g}}_{i}^{\mu },\sigma_{g}^{2}\right)+1\times \int_{\theta }^{+\infty }dgf\left(g|{\bar{g}}_{i}^{\mu },\sigma_{g}^{2}\right)\\ \;=-F\left(\theta |{\bar{g}}_{i}^{\mu },\sigma_{g}^{2}\right)+1-F\left(\theta |{\bar{g}}_{i}^{\mu },\sigma_{g}^{2}\right)\\ \;=\text{ erf}\left(\frac{{\bar{g}}_{i}^{\mu }-\theta }{\sqrt{2}\sigma_{g}}\right), \end{array}$$

where $f\left(g|{\bar{g}}_{i}^{\mu },\sigma_{\mu }^{2}\right)$ and $F\left(g|{\bar{g}}_{i}^{\mu },\sigma_{\mu }^{2}\right)$ are probability density function and cumulative density function, respectively, of a normal distribution with mean ${\bar{g}}_{i}^{\mu }$ and variance $\sigma_{g}^{2}$. To arrive at the desired target output, e.g., $\eta =\text{sgn}\left(\overset{N}{\underset{i=1}{\sum }}W_{i}S_{i}^{\mu }\right)$ for all μ, the cost function

$$\begin{array}{l} E=\overset{P}{\underset{\mu =1}{\sum }}{\left(\eta^{\mu }-W^{T}{\bar{S}}^{\mu }\right)}^{2} \end{array}$$

should be minimized. We then find the linear least squared error solution to *W*,

(7) $$\begin{array}{l} W^{T}=\underset{\tilde{W}}{\text{argmin}}\left[\overset{P}{\underset{\mu }{\sum }}{\left(\eta^{\mu }-{\tilde{W}}^{T}{\bar{S}}^{\mu }\right)}^{2}\right], \end{array}$$

by taking the Moore-Penrose pseudoinverse of the matrix $\bar{S}$,

(8) $$\begin{array}{ll} W^{T} & =\eta {\bar{S}}^{*} \end{array}$$

(9) $$\begin{array}{l} =\eta V\Sigma^{*}U^{T}\;, \end{array}$$

where S^*^ is the pseudoinverse of *S*, *U* and *V* are the matrices of left and right singular vectors, respectively, and Σ is the matrix of singular values. Here the Moore-Penrose pseudoinverse enables us to look for the best approximation using column vectors of ${\bar{S}}_{i}^{\mu }$. The approximation is also the best-fit solution minimizing the cost function. More explanation about Moore-Penrose pseudoinverse can be found in Appendix A.

**Figure 1:**
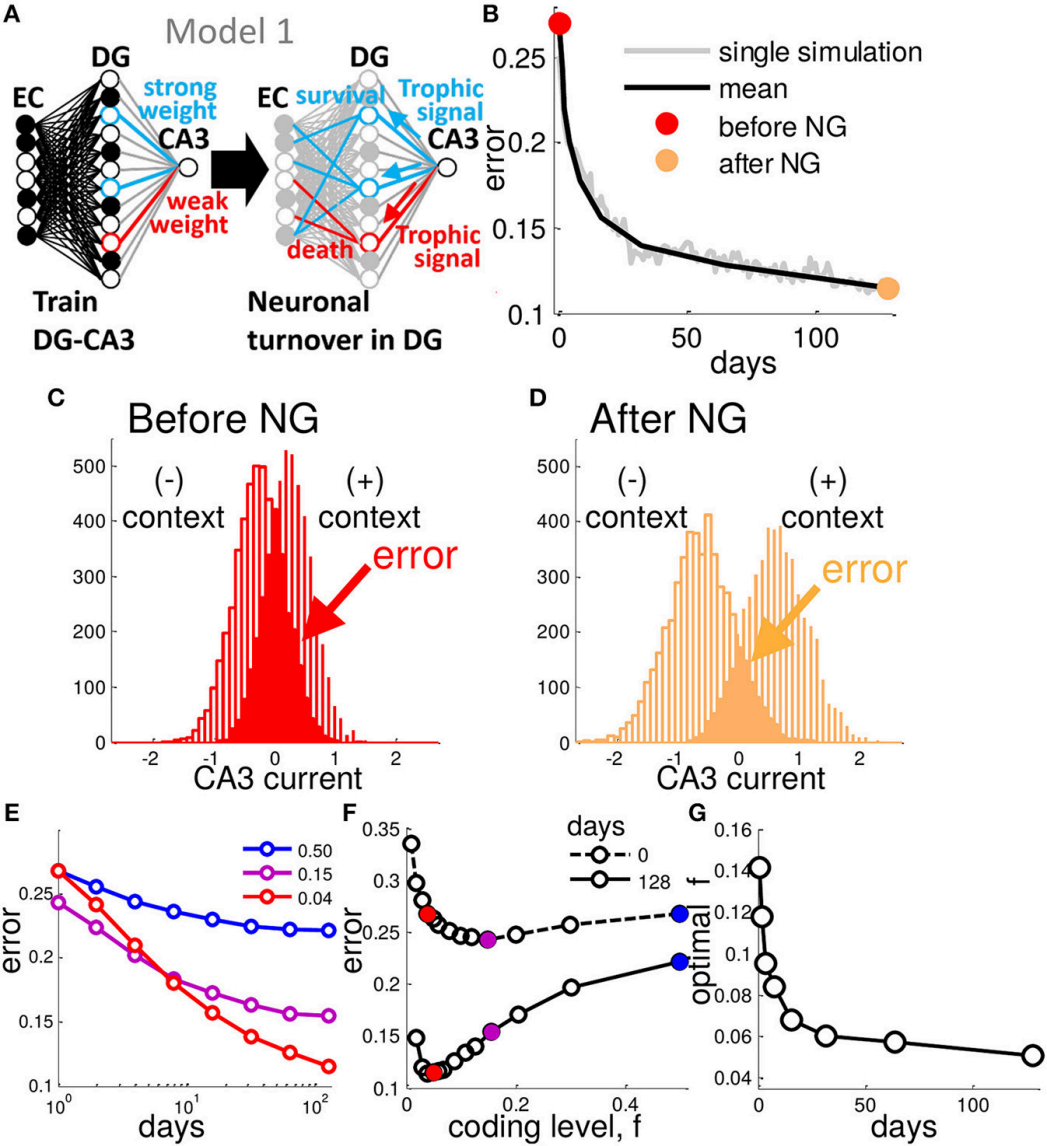
Neurogenesis enhances generalization performance. **(A)** In Model 1, after a weight vector is assigned by training, DG units with weak weights to CA3 are replaced with new randomly connected units. **(B)** At each day of training the network is tested with randomly generated patterns belonging to one of the two contexts. This generalization error decreases as a function of the number of iterations of neural turnover. Single simulation (gray) and mean of many simulations (black), before (red point) and after (orange point) neurogenesis. **(C,D)** CA3 Synaptic current distribution for all test patterns representing the two contexts before **(C)** and after **(D)** 128 iterations (days) of neural turnover. Results are from a network of 200 EC, 500 DG neurons and a single CA3 readout. Each context consists of 50 EC patterns with input noise, ν, fixed at 0.2, and theta is chosen to yield a coding level of *f* = 0.04, turnover rate is fixed at 0.30 (See Experimental Procedures). **(E)** Mean error is shown decreasing as a function of the number of iterations of neural turnover for three different coding levels. **(F)** Error is shown as a function of coding level before and after 128 iterations of neural turnover. After neurogenesis the performance is improved at all levels of sparseness (all coding levels, *f*). **(G)** The coding level at which minimum error occurs (optimal *f*) is plotted vs. the number of iterations of neural turnover. Neural turnover favor a sparser (reduced) coding level. Mean error is calculated as the mean of 20 simulations.

**Figure 2:**
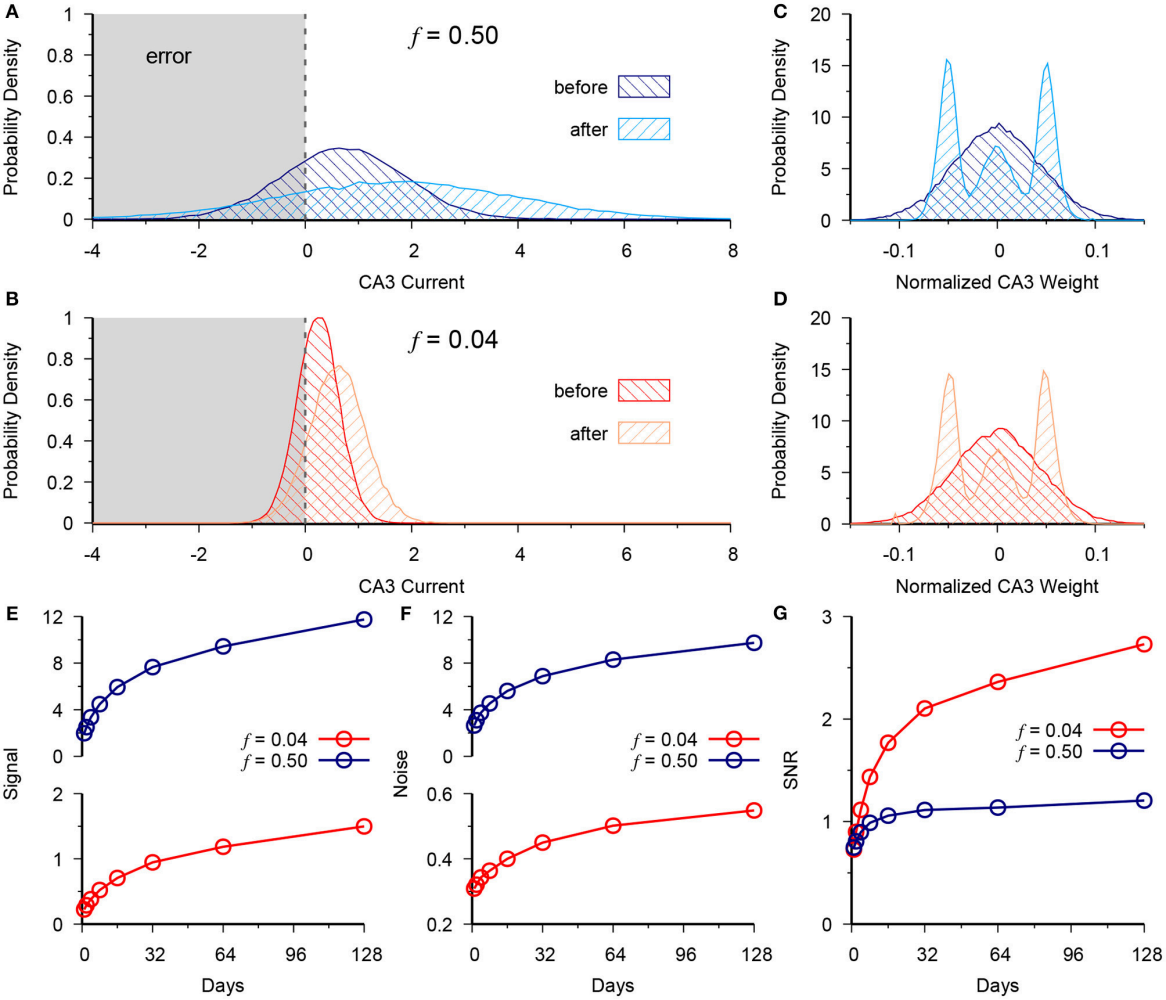
Neurogenesis exploits the low noise of the sparse code to outperform dense DG coding. **(A)** Distribution of CA3 current at *t* = 0 (before) vs. *t* = 128 (after) for the dense activity case of *f* = 0.5 for a group of test patterns generated from a single prototype pattern belonging to the (+) context. Vertical dashed line at 0 represents the activity threshold of the CA3 neuron **(B)** Same as in **(A)**, but for the sparse case of *f* = 0.04. **(C,D)** Normalized CA3 readout weight distribution in dense **(C)** and sparse **(D)** cases. **(E)** Signal at CA3 vs. time for *f* = 0.5 (blue) and *f* = 0.04 (red). **(F)** Readout noise at CA3 vs. time for *f* = 0.5 (blue) and *f* = 0.04 (red). **(G)** Signal to noise ratio (SNR), calculated as data in **(E)** over data in **(F)**. Demonstrates the advantage given by slower scaling of variance in the sparse case of *f* = 0.04. The results are plotted as the mean of 20 simulations.

**Figure 3:**
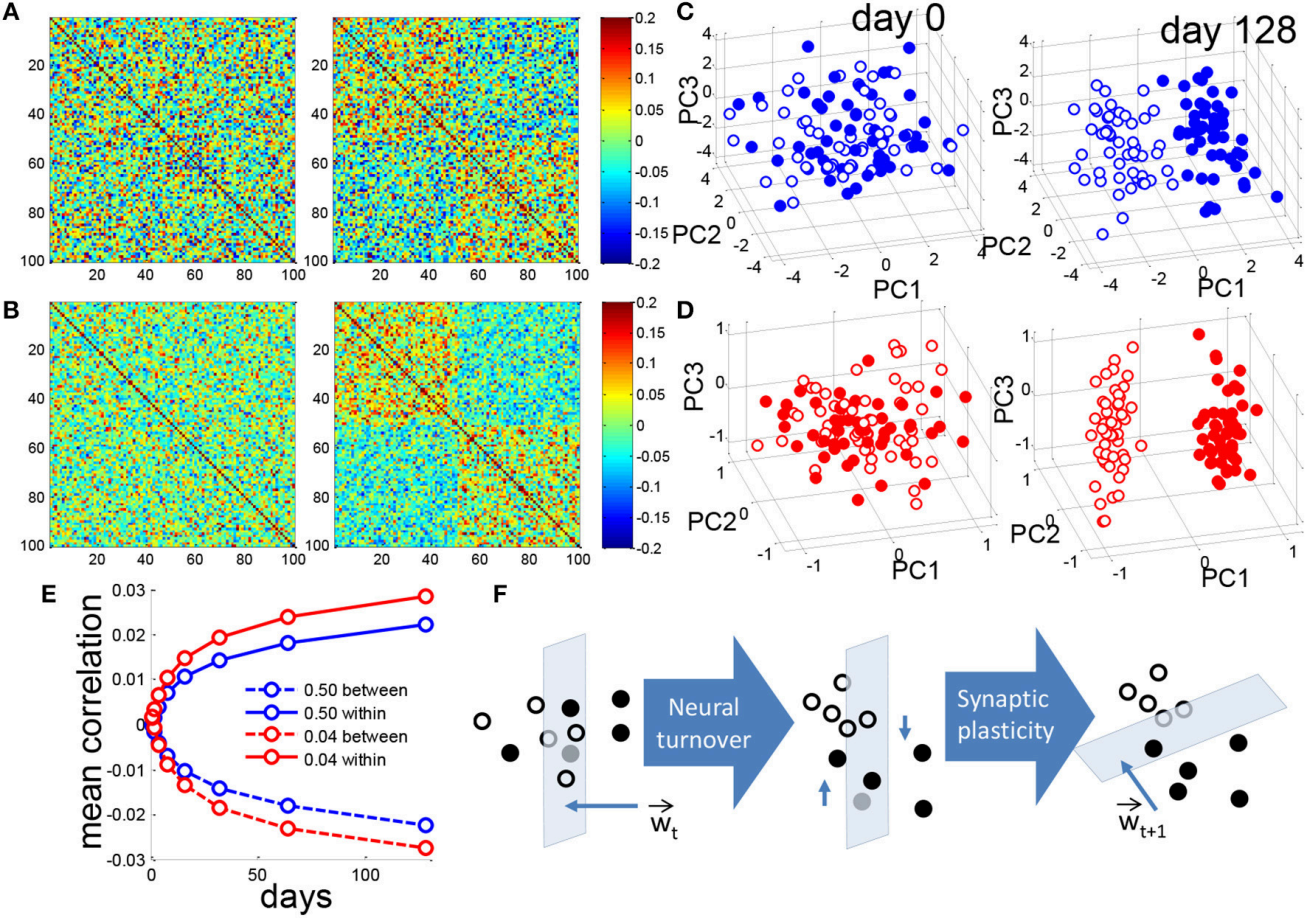
Neurogenesis clusters context representations in DG activity space. **(A)** Matrix of pairwise correlations between training patterns represented in the DG, ordered by context so that patterns 1–50 correspond to the (+) context and patterns 51–100 correspond to (-) context. For a single simulation the correlation matrix of patterns for *f* = 0.50 before (left) and after (right) 128 iterations of neural turnover. **(B)** Same as in **(A)** but for *f* = 0.04. **(C)** Training patterns from the two contexts are projected onto the principal components. For visual clarity only the means of all training patterns for each of the 100 prototypes are projected. Closed and open circles correspond to the (+) and (-) contexts, respectively. Dense coding, *f* = 0.50, before (left) and after (right) 128 iterations of neural turnover. **(D)** as in **(C)** but for sparse coding of *f* = 0.04. **(E)** Mean correlation between patterns of opposite contexts (between) and patterns of the same context (within), calculated as mean of 20 simulations. **(F)** Schematic illustration of context discrimination by neurogenesis. Closed and open circles represent the patterns of the two respective contexts. Intuitively, as neuronal turnover and retraining proceeds the patterns in DG space are shifted in dimensions that are mostly parallel to the weight vector, over time leading to greater separation. All above results are from a single simulation.

To implement the synaptic competition underlying neurogenesis we compare three different models. In Model 1 (Figures 1, 2, 3, **5**), at each time step we kill DG units corresponding to the bottom 30% of absolute values in vector *W*, i.e., the input weights to those units are re-randomized. In Model 2 we explore a multicontext case presented in Figure **6** in which each DG unit projects to multiple CA3 units, therefore we take the sum of the absolute value of each DG units weight vector and compare this value across all DG units.

In Model 3 presented in **Figure 7**, rather than re-randomizing all input weights of selected DG units, we determine the probability of synaptic turnover of each DG unit from a linear transfer function of its DG-CA3 weight (Figure S3A). Results presented in **Figure 7** are from the mean of 100 simulations with a slope = 2.5 for the linear transfer function.

### 2.3. Analyzing Performance of the Network

To evaluate the performance of the network, the signal-to-noise ratio is introduced. The signal is defined by the square of the expectation of the difference between CA3 synaptic currents corresponding to (+) context, i.e., η^μ^ = +1, and (−) context, i.e., η^μ^ = −1, among all the patterns.

(10) $$\begin{array}{l} \text{Signal}={\left[\text{E}\left(h^{\mu }|\eta^{\mu }=+1\right)-\text{E}\left(h^{\mu }|\eta^{\mu }=-1\right)\right]}^{2}. \end{array}$$

To progress, we define the context-bias of a given DG unit *i*, Ψ_*i*_, as the difference between the fraction of (+) context patterns, $f_{i}^{+}$, and the fraction of (−) context patterns, $f_{i}^{-}$, to which it responds.

(11) $$\begin{array}{l} {\text{Ψ}}_{i}=f_{i}^{+}-f_{i}^{-}, \end{array}$$

where $f_{i}^{\pm }$ is the fraction of (±) context patterns activating DG unit *i*. On the other hand,

(12) $$\begin{array}{l} {\left(S\eta^{T}\right)}_{i}=\underset{\text{active for a }(+)\text{ pattern}}{\sum }\left(+1\right)\times \left(+1\right)\\ \;+\underset{\text{inactive for a }(+)\text{ pattern}}{\sum }\left(-1\right)\times \left(+1\right)\\ \;\underset{\text{active for a }(-)\text{ pattern}}{\sum }\left(+1\right)\times \left(-1\right)\\ \;+\underset{\text{inactive for a }(-)\text{ pattern}}{\sum }\left(-1\right)\times \left(-1\right) \end{array}$$

(13) $$\begin{array}{l} =Pf_{i}^{+}-P\left(1-f_{i}^{+}\right)-Pf_{i}^{-}+P\left(1-f_{i}^{-}\right) \end{array}$$

(14) $$\begin{array}{l} =2P{\text{Ψ}}_{i}\;. \end{array}$$

Note that *S* here is a matrix, whose column vectors are activities of DG neurons for different input patterns. η is a label vector, where entries are expected output (CA3) of the patterns. Then we can then express the context-bias in terms of *S* and η in a matrix-vector equation as:

(15) $$\begin{array}{l} \text{Ψ}=\frac{1}{2P}S\eta^{T}\;. \end{array}$$

With this, the signal can be expressed as

(16) $$\begin{array}{ll} \text{Signal} & ={\left[2\underset{i}{\sum }W_{i}\left(f_{i}^{+}-f_{i}^{-}\right)\right]}^{2} \end{array}$$

(17) $$\begin{array}{l} ={\left[2W^{T}\text{Ψ}\right]}^{2}\;. \end{array}$$

On the other hand, we define the noise as the sum of variances of this current for the (+) and (−) contexts respectively:

(18) $$\begin{array}{ll} \text{Noise} & =\text{Var}\left(h^{\mu }|\eta^{\mu }=+1\right)+\text{Var}\left(h^{\mu }|\eta^{\mu }=-1\right) \end{array}$$

(19) $$\begin{array}{ll} \overset{N\to \infty }{=} & 4\underset{i}{\sum }W_{i}^{2}\left[f_{i}^{+}-{\left(f_{i}^{+}\right)}^{2}+f_{i}^{-}-{\left(f_{i}^{-}\right)}^{2}\right] \end{array}$$

(20) $$\begin{array}{l} =4\underset{i}{\sum }W_{i}^{2}\left[f_{i}^{+}\left(1-f_{i}^{-}\right)+f_{i}^{-}\left(1-f_{i}^{+}\right)-{\left(f_{i}^{+}-f_{i}^{-}\right)}^{2}\right] \end{array}$$

From these expressions we derive the signal to noise ratio (SNR) in terms of $f_{i}^{\pm }$ and *W*^*T*^.

(21) $$\begin{array}{l} \frac{\text{Signal}}{\text{Noise}}=\frac{{\left[2W^{T}\text{Ψ}\right]}^{2}}{4\sum_{i}W_{i}^{2}\left[f_{i}^{+}\left(1-f_{i}^{-}\right)+f_{i}^{-}\left(1-f_{i}^{+}\right)-{\left(f_{i}^{+}-f_{i}^{-}\right)}^{2}\right]} \end{array}$$

(22) $$\begin{array}{l} =\frac{\sum_{i}W_{i}^{2}{\text{Ψ}}_{i}^{2}+\sum_{i\neq j}W_{i}{\text{Ψ}}_{i}W_{j}{\text{Ψ}}_{j}}{\sum_{i}W_{i}^{2}\left[f_{i}^{+}\left(1-f_{i}^{-}\right)+f_{i}^{-}\left(1-f_{i}^{+}\right)-{\left(f_{i}^{+}-f_{i}^{-}\right)}^{2}\right]} \end{array}$$

(23) $$\begin{array}{ll} \overset{N\to \infty }{=} & \frac{\sum_{i}W_{i}^{2}{\left(f_{i}^{+}-f_{i}^{-}\right)}^{2}}{\sum_{i}W_{i}^{2}f_{i}^{+}\left(1-f_{i}^{-}\right)+\sum_{i}W_{i}^{2}f_{i}^{-}\left(1-f_{i}^{+}\right)-\sum_{i}W_{i}^{2}{\left(f_{i}^{+}-f_{i}^{-}\right)}^{2}} \end{array}$$

This expression allows us to observe the intuitive relationship between the context-bias of DG cells and the SNR. The second term in the numerator of Equation (22) should vanish as *N* → ∞, as it sums random numbers centered at zero.

For *W*^*T*^,

(24) $$\begin{array}{l} W^{T}=2P{\text{Ψ}}^{T}U{\left(\Sigma^{*}\right)}^{2}U^{T} \end{array}$$

In the presentation of our results it is useful to let ${\hat{\text{Ψ}}}_{i}^{T}={\text{Ψ}}^{T}u_{i}u_{i}^{T}$ where *u*_*i*_ is the *i*-th column of *U*, so that

(25) $$\begin{array}{l} W^{T}=2P\overset{N}{\underset{i}{\sum }}\sigma_{i}^{-2}{\hat{\text{Ψ}}}_{i}^{T} \end{array}$$

observing the weight vector as a weighted sum of projected context-bias vectors. The derivation of this equation can be found in Appendix B.

### 2.4. Dimensionality of DG Contextual Representation

From above, the weight vector is defined as:

(26) $$\begin{array}{l} W^{T}=\eta V\Sigma^{*}U^{T} \end{array}$$

Permitting us to rewrite the weight vector as a linear sum of coefficients producted with their respective left singular vectors:

(27) $$\begin{array}{l} W=\overset{D}{\underset{i=1}{\sum }}\alpha_{i}u_{i}, \end{array}$$

where *D* is the dimension of the square matrix *U*. The *D* dimensions are ranked from 1 to *D* according to their corresponding coefficients. We define a cumulative weight vector of a given dimensionality as:

(28) $$\begin{array}{l} {Ŵ}_{d}=\overset{d}{\underset{i}{\sum }}\alpha_{i}u_{i}\;, \end{array}$$

where *d* takes a value from 1 to *D*, representing the number of dimensions chosen for a given cumulative weight vector. We then define the cumulative performance, ${\text{perf}}_{\text{cum}}=\frac{\left(0.5-\text{err}\right)}{0.5}$, where the error is calculated for every cumulative weight vector (Figure S3C).

### 2.5. Model Parameters

**Figure 4:**
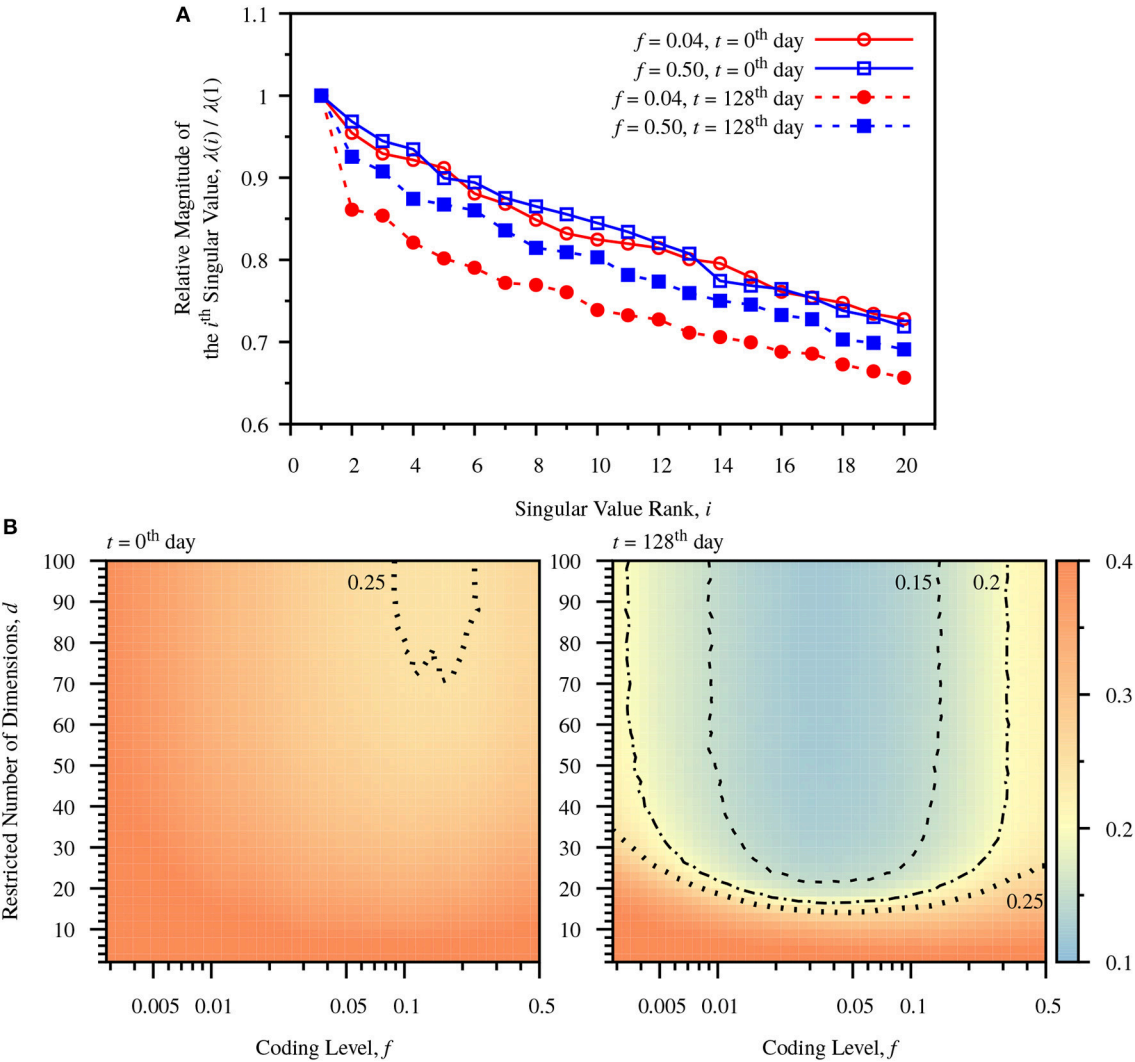
Dimensionality reduction due to neurogenesis. **(A)** Relative magnitudes of ranked singular values, λ(*i*)/λ(1). The singular values are calculated for the centered DG activity matrix for a single simulation. In both cases the relative magnitudes of singular values drop after turnover of DG neurons. The sparse case (*f* = 0.04) shows larger drops than the dense case (*f* = 0.50). **(B)** Color-maps of classification error comparing predefined coding level, *f*, and restricted dimension *d* at different times, *t* = 0th day and *t* = 128th day. The number of dimensions used to calculate *W* is restricted to *d*, according to Equation (28). The error is the average error measured from 20 simulations. Before neuronal turnover, the map is relatively flat. After neuronal turnover there is a large region of low dimensionality over which the classification performance of the network maintains low error. Dashed line: contour for err = 0.15. Dot-Dashed Curve: contour for err = 0.20. Dotted line: contour for err = 0.25.

**Figure 5:**
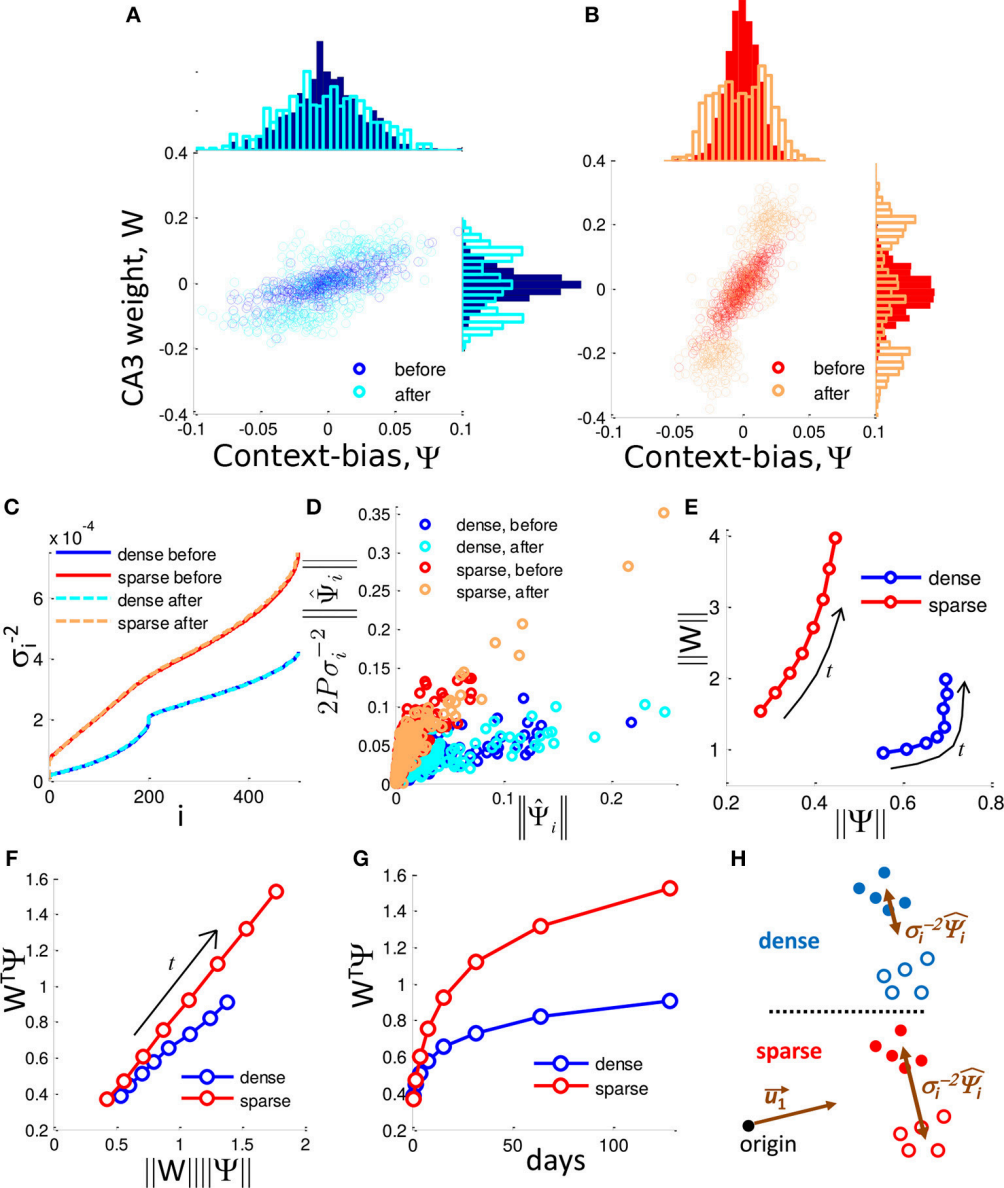
Selection of context-biased DG units takes advantage of the singular value distribution of the sparse code. **(A,B)** DG-CA3 weight vs. context-bias of individual DG neurons before and after neurogenesis for *f* = 0.50 **(A)** and *f* = 0.04 **(B)**. Marginal histograms show the projected distributions. In both cases the DG-CA3 weights and the context-bias of DG neuron evolve to a bimodal distribution in which they are correlated. **(C)** Inverse square singular values, $\sigma_{i}^{-2}$, sorted by index, *i*. **(D)** The influence of the context-bias vector on the weight vector is determined by the relationship between $2P\sigma_{i}^{-2}\|{\hat{\text{Ψ}}}_{i}\|$ and $\left\|{\hat{\text{Ψ}}}_{i}\right\|$ over time. Plot shows the dense case (*f* = 0.50) and sparse case (*f* = 0.10) before and after neuronal turnover (128 iterations). **(E)** ‖*W*‖ grows more rapidly as a function of ‖Ψ‖ in the sparse case. Arrows label the direction of evolution. **(F)** *W*^*T*^Ψ grows more rapidly in the sparse case than in the dense case as a function of the product ‖*W*‖‖Ψ‖. Arrow labels the direction of evolution. **(G)** *W*^*T*^Ψ grows more rapidly in time in the sparse case, and determines the scale up of the SNR. All results are calculated from a single simulation. **(H)** Dense coding (blue, top) results in a reduced contribution of separating components, $\sigma_{i}^{-2}$ while sparse coding (red, bottom) results in less reduction in the contribution of these components, promoting greater separation of contexts in DG activity space.

**Figure 6:**
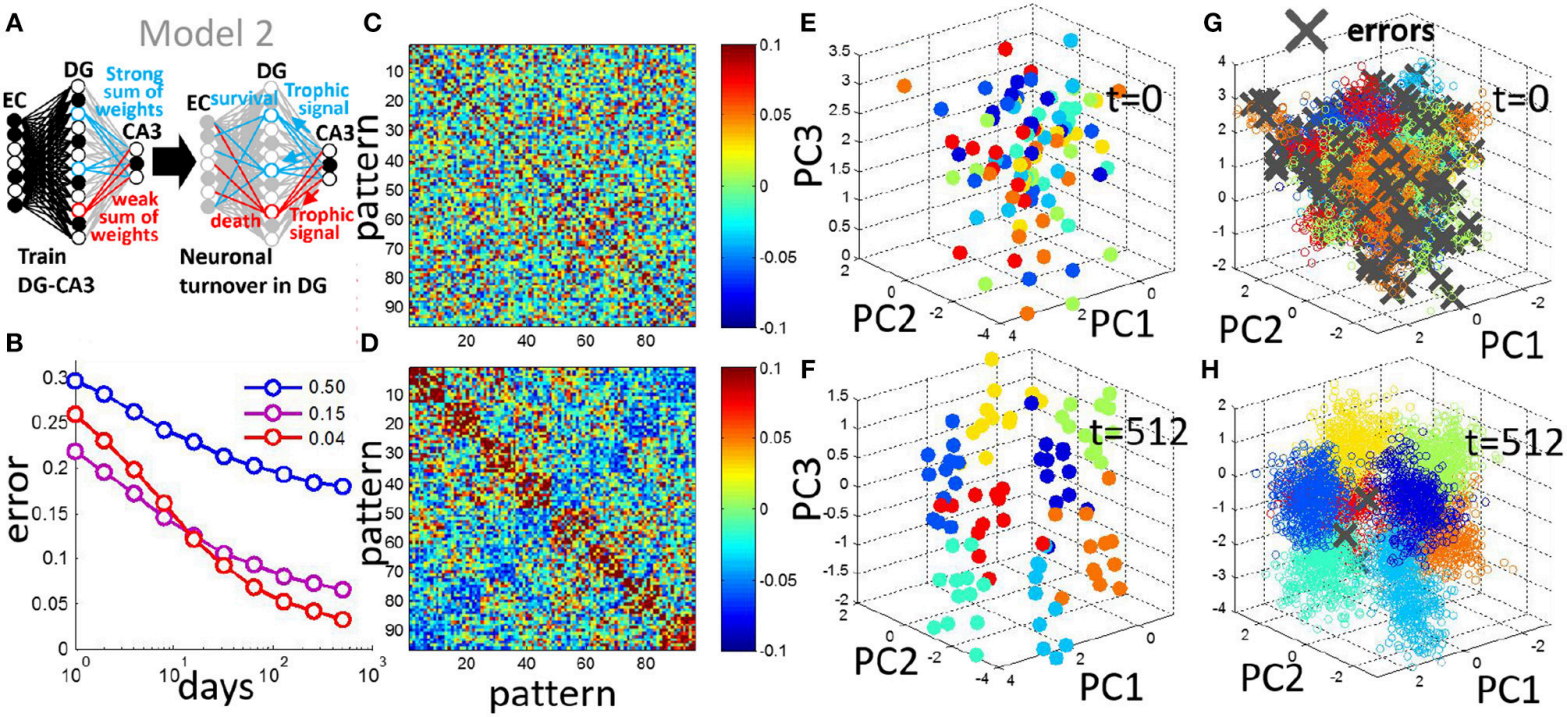
Neuronal turnover rule can be generalized to encode multiple contexts. **(A)** In Model 2, multiple context discrimination is performed by using multiple readout units each with trained weights. The turnover rule sums the absolute readout weights of all units and eliminates the units ranking in the bottom 30%. **(B)** Generalization error decreases with neurogenesis, and the sparse code is optimal for the multicontext case, shown as the mean of 20 simulations (input noise, ν = 0.05, 12 prototypes per context). **(C)** For a single simulation, pairwise correlation matrix of patterns in DG space before neurogenesis. **(D)** Same as in **(C)** after 512 days of neurogenesis. Patterns evolve into correlated groups in DG space. **(E)** Projection of patterns in DG space onto PCs, before neurogenesis. **(F)** Same as in **(E)** after 512 iterations of neurogenesis. Clusters emerge from a random arrangement, and move apart from each other. **(G)** As in **(E)** but projection of test patterns onto PCs, day 0 before neurogenesis. **(H)** as in **(G)** but after day 512 of neurogenesis. Patterns representing distinct contexts cluster together, and become separated from each other.

**Figure 7:**
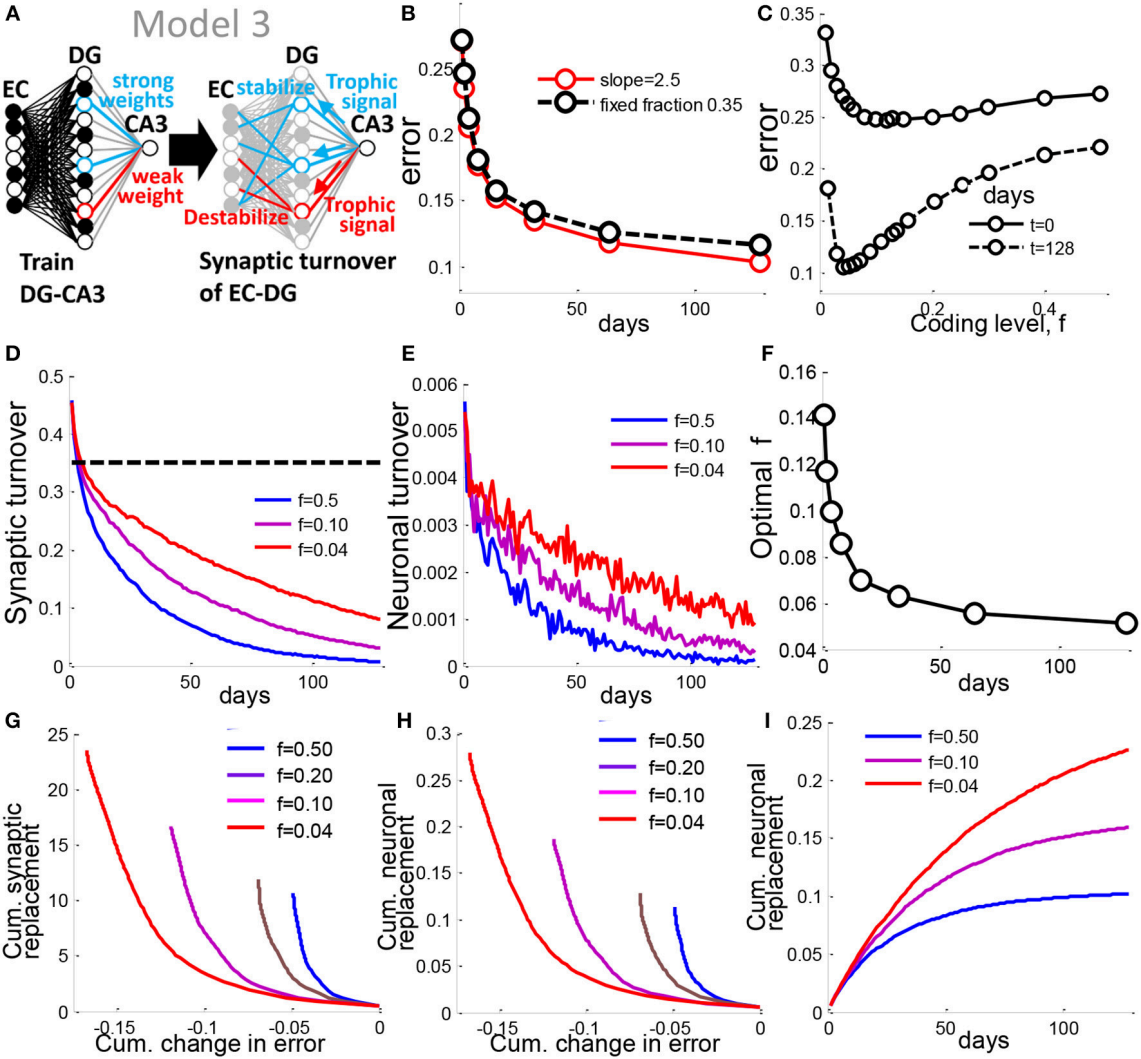
A synaptic turnover rule generalizes neuronal turnover to allow prediction of biological rates. **(A)** In Model 3, the strength of a DG neurons weight to CA3 is used to determine the probability of turnover of EC-DG synapses onto that neuron. **(B)** Error vs. time for synaptic turnover model with slope set to 2.5, is similar to Model 1 in which a fixed fraction of 0.30 DG units are turned over. **(C)** the optimal coding level is between 4 and 5% as in the prior model. **(D)** Fraction of synapses turned over as a function of time for different coding levels, f. The sparsely coded DG requires greater synaptic turnover. Yet Model 3, for all f, requires less turnover than Model 1 (dotted black line) for a similar level of performance. **(E)** Fraction of neurons turned over vs. time. The sparse case requires more DG units to be turned over. **(F)** For each time point, the coding level at which optimal performance is achieved is evaluated, and plotted as optimal coding level. The optimal coding level becomes more sparse in time as in Model 1 and 2. **(G)** The tradeoff between cumulative synaptic turnover vs. cumulative reduction in error is best resolved by the sparse DG. **(H)** same as in G but for neural turnover. **(I)** Cumulative neuronal replacement of DG vs. time, corresponding well with experimental data suggesting around 10% of the mature DG is replaced by adult-born cells (Imayoshi et al., 2008). All results are calculated as the mean of 100 simulations, with slope = 2.5 for the linear transfer function (See Experimental Procedures). See also Figure S1.

All results in Figures 1–**5** are from a network with 200 EC, 500 DG units and a single CA3 unit. Data in **Figure 6** are from the same size network except that the number of CA3 units is increased to 3 to allow for multicontext discrimination. In Figures 1, 2, 3, 4, **7** the network was trained with the mean representation of each of 100 prototype patterns as described above. In **Figure 6** the network was trained with 8 groups of 12 prototypes, to represent 8 subcontexts, by calculating the mean representation of each prototype assuming some variability as described above. In Figure 5 the network is the same size, however, training consisted of 100 noisy instances of 100 prototypes, rather than using the mean representation of each prototype. This is because we wished to relate the results of this training directly to the equations that we derived for the SNR from the SVD as above. Both types of training gave similar qualitative results, therefore they are not explicitly compared.

## 3. Results

### 3.1. Network Model for Adult Neurogenesis in the Formation of Associative Memories

We implement a feed-forward multilayer perceptron in which pattern discrimination (classification) is the readout of performance. The model consists of a three-layer network including entorhinal cortical inputs (EC), dentate gyrus (DG), and a CA3 output (Figure 1A). We assume that a given DG cell receives a weighted sum of its inputs from the EC. Thus the total current into the *i*'th DG cell in response to the μ'th EC pattern, ξ^μ^, is given by $g_{i}^{\mu }=\sum_{j}^{M}J_{j}\xi_{j}^{\mu }$ where the weights, *J*_*j*_, are drawn randomly from a normal distribution, N(0,1), and its activity is determined by the nonlinear function of this current, $S_{i}^{\mu }=\text{sgn}\left(g_{i}^{\mu }-\theta \right)$, where we refer to θ as the activation threshold, which is a tunable parameter we use to control the coding level, i.e., the expected value of the fraction of patterns to which a given unit responds, defined as $f=\frac{1}{2P}\overset{P}{\underset{\mu =1}{\sum }}\frac{1}{N}\overset{N}{\underset{i=1}{\sum }}\left(S_{i}^{\mu }+1\right)$. We define a context as a group of prototypical activity patterns generated in the EC, where each pattern represents a stimulus that is present in the given context. We assume that there is random variability in the environment, or within the system such that among these patterns each binary element may be flipped with probability ν. Averaging for each prototype over the input noise ν, we obtain corresponding mean input currents for each DG cell for each prototype pattern, ${\bar{g}}_{i}^{\mu }=g_{i}^{\mu }\left(1-2\nu \right)$, with variance $\sigma_{g}^{2}=4M\nu \left(1-\nu \right)$ (See Materials and Methods). This gives us a set of mean prototype activity patterns in the space of DG activity, where each neurons activity is defined as $\bar{S}=\text{erf}\left[\left(\theta -{\bar{g}}_{i}^{\mu }\right)/\left(\sqrt{2}\sigma_{g}\right)\right]$. The network is said to perform contextual discrimination when the CA3 output correctly reads out the DG patterns according to the target label for the EC context to which those patterns belong.

To train the network, we randomly assign to the μ-th EC pattern, a CA3 target, η^μ^, taking the value +1 or −1. Thus, assuming that θ is held constant during training, the task of the network is to find a input weight matrix, *J*, and an output weight vector, *W*, such that $W^{T}\bar{S}=\eta$, where $\bar{S}$ is the matrix of DG prototype patterns, and η is the corresponding vector of context labels.

We hypothesize that neurogenesis provides a mechanism by which biology breaks this problem into two steps. We assume that, as in the brain, the time-scale of neurogenesis is much slower than that for synaptic plasticity, allowing us to train the output weights, *W*, independently of the input weights, *J*. Many learning rules could be used to train *W*, such as Hebbs rule, or Support Vector Machine (with a linear kernel), or Linear Discriminant Analysis. We obtained qualitatively similar results with all of these, therefore, to simplify later analysis, we use the pseudoinverse rule yielding $W^{T}=\eta {\bar{S}}^{*}$, where ${\bar{S}}^{*}$ is the Moore-Penrose pseudoinverse of the matrix of DG prototype patterns, and *W*^*T*^ is the transpose of the output weight vector, *W*, whose elements are the DG-CA3 weights of the population of DG units (See Materials and Methods). Next we assume that DG neurons compete with each other for connection to CA3 such that the absolute value of *W*_*i*_ determines their probability of survival, i.e., neurons with large values will receive some trophic signal allowing them to survive, while those with values below some threshold will die, to be replaced by a new randomly connected unit (Figure 1A). Thus training is summarized as follows:

1. Initialize the matrix of random EC-DG weights, *J*.
2. Calculate DG-CA3 weight vector, *W*, by $W^{T}=\eta {\bar{S}}^{*}$.
3. Eliminate DG units with the weakest |*W*_*i*_|s at a predefined percentage (to be stated in the following).
4. Those DG units are replaced by new DG units. The EC-DG weights connecting to those new DG units are randomly drawn from a normal distribution N(0,1).
5. Repeat and start from Step 2.

Since the cell cycle in biology corresponds to about 24 h, and each iteration of our model represents the death and birth of neurons, one iteration corresponds to roughly one biological day (the time axes is labeled “days”). One should note that the DG neurons considered in this model are those mature enough to emerge into the dentate gyrus and reach CA3. Those immature adult-born cells unable to reach CA3 are not considered in this model.

We test the network by presenting EC input patterns with a fraction of ν bits flipped (corresponding to input noise, or variability) that belong to a known context, taking the CA3 output for the μ-th test pattern as ${\hat{\eta }}^{\mu }=\text{sgn}\left(\overset{N}{\underset{i}{\sum }}W_{i}S_{i}^{\mu }\right)$, where *N* is the total number of DG units. Then we measure the error on a given test pattern, ${\text{err}}^{\mu }=\{\begin{array}{c} 0,\;\text{if}\;{\hat{\eta }}^{\mu }=\eta^{\mu }\\ 1,\;\text{otherwise} \end{array}$, and mean over all test patterns, ${\langle {\text{err}}^{\mu }\rangle }_{\mu }$, yielding the generalization error. Neuronal turnover of the weakest 30% of DG neurons per day results in a steadily decreasing mean error as a function of the number of iterations (days) of contextual associative learning (Figure 1B), thus increasing the performance of this framework relative to the randomly initialized network corresponding to the case studied by Barak et al. (2013) and Babadi and Sompolinsky (2014). The choice of 30% may seem arbitrary, but further clarification will follow. The error in Figure 1B is determined by the overlap between the two underlying distributions of total synaptic current into CA3 for the two contexts in the presence of variability on the input (Figure 1C). The sign of the CA3 readout should be opposite for each of the two possible associations, positive or negative for a given pattern belonging to the context with (+1) or (−1) context, respectively. After 128 days of neural turnover the spread between the distributions increases such that the overlap between them, is decreased (Figure 1D). From Figure 1B we see that the initial drop in error occurs rapidly, i.e., most of the performance gain from neurogenesis occurs within a week.

### 3.2. Neurogenesis Interacts With Sparse Activity to Enhance Contextual Discrimination

Sparseness of granule cell firing is likely induced via a combination of cell-intrinsic and extrinsic properties (Marin-Burgin et al., 2012). We control sparseness by adjusting θ which represents the combination of these effects, determining the cells coding level, *f*. Neurogenesis increases performance at all coding levels (Figures 1E,F). The optimal code becomes mores sparse and appears to plateau at around 4–5% of DG cells active (Figure 1G). Thus, in contrast to the initial optimal coding level of around 10–15% active, similar to previous reports in a similar framework (Barak et al., 2013; Babadi and Sompolinsky, 2014), our best performance is achieved at a very sparse activity level that continues to sparsen with time (Figure 1G).

The error reduction depends on the turnover rate, i.e., the fraction of neurons targeted for turnover per day (Figure S1A), such that longer periods of learning (more iterations of neurogenesis) favored lower turnover rates (Figure S1B). On average, the optimal rate of turnover is a monotonically decreasing function of the number of days learning (Figure S1C), yielding an optimal turnover rate of around 0.3 at 128 days of learning.

We next analyzed the dynamics of the population of DG neurons. The survival rate of neurons during the time course of encoding the contexts depended on their age, i.e., those born more recently have a survival advantage (Figure S1D), indicating the gradual replacement of existing cells with those that are newly born. Neuronal replacement is highest at the beginning of learning, with a fraction of around 0.7 of 1-day old neurons surviving, but after 256 days of learning even 1-day old neurons survive at a very low rate of around 0.04. Whenever there is a sudden change of the contexts in the 2-class case, or addition of a context in the multiclass case, we would indeed see a sudden jump in the survival rate of newborn neurons. Therefore the survival rate of newborn neurons scales with the learning rate, or the encoding of new information, consistent with experimental findings (Kempermann et al., 1997a,b; Gould et al., 1999).

To explore the relationship between connectivity and sparse coding, we tested the networks performance for varying degrees of connectivity from EC to DG (Figure S2A). The performance degrades as input connectivity is reduced, with the performance of sparsely coded models suffering more than that of more densely coded models (Figures S2A,B). Nevertheless the optimal coding level is a steeply monotonically decreasing function of the connectivity that is sparse above a connectivity of around 2.5% (Figure S2C), suggesting that sparse models perform well over a large range of connectivities.

We next analyzed the CA3 readout to determine why the memory performance scales up more quickly in the sparse vs. the dense coding case as a function of neurogenesis. We observe the total synaptic current coming into CA3 from the DG for a single test pattern that belongs to the (+) context. Accordingly we see that neurogenesis causes a positive shift in the distribution of total synaptic current into CA3 for both the dense (Figure 2A) and sparse (Figure 2B) cases with the normalized output weights shown in panels C and D, respectively. However, there is an accompanying increase in the spread of this distribution countering the performance gain given by the increased signal, since the tail of the distribution causes errors when it crosses the CA3 decision boundary (Figure 2A).

We define the Signal to Noise ratio (SNR) as:

(29) $$\begin{array}{l} \begin{array}{l} \frac{\langle \text{Signal}\rangle }{\langle \text{Noise}\rangle }\overset{\text{def}}{=}\frac{{\langle h_{+}-h_{-}\rangle }^{2}}{\langle \sigma_{h_{+}}^{\;2}\rangle +\langle \sigma_{h_{-}}^{\;2}\rangle }, \end{array} \end{array}$$

where *h*_+_ and *h*_−_ are the total synaptic current into CA3 from the patterns of the (+) and (−) contexts, respectively, and $\sigma_{+}^{2}$ and $\sigma_{-}^{2}$ are the respective variances of that current across patterns. In both the dense and sparse cases neurogenesis contributes to a scale-up of the signal (Figure 2E) and the noise (Figure 2F). Yet, in the signal-to-noise ratio (SNR) we see the superior performance of the sparse case (Figure 2G). Due to synaptic competition, the distribution of DG-CA3 weights gradually shifts to higher efficacy synapses for both the dense (Figure 2C) and the sparse case (Figure 2D).

### 3.3. Neurogenesis, Synaptic Plasticity, and Sparse Activity Cooperatively Facilitate Dimensionality Reduction

We then ask how the representation in the DG changes over time. Prior to neurogenesis there is no correlation among the patterns representing the two contexts for either the dense or sparse case (Figures 3A,B, left). After neurogenesis proceeds, for both the dense and sparse case, patterns that belong to a given context become correlated to each other, while those that belong to different contexts become anticorrelated (Figures 3A,B, right). Note that for the same amount of neural turnover, the sparse case always achieves a more correlated representation (Figure 3E). Figure 3E shows the mean correlations within the same context and across different contexts shown in panels A and B. It suggests that the representations in DG for different patterns in the same context are similar, while representations for patterns in different contexts are more different after training.

For a closer look of the representations before and after the neurogenesis training, Principal Components Analysis (PCA) was used for presentations. Principal Components Analysis reveals that, initially the DG activity patterns are randomly distributed (Figures 3C,D, left) but after neurogenesis proceeds, patterns representing the two contexts become clustered, and separated, for both the dense and the sparse case (Figures 3C,D, right), while the sparse case clearly shows greater separation along PC1 (Figure 3D, right). Note that, though we do not show it here, the separation between clusters became observable after only 10–15 days. Since it becomes clearer with a long simulation time, we report the state at the 128th day for comparison.

We intuitively illustrate the effect of neuronal turnover (Figure 3F). Synaptic plasticity, between the DG and CA3, assigns a weight vector at a given time, *t*, *W*_*t*_. This weight vector defines a perpendicular hyperplane that separates the patterns defining the two contexts from each other in the space DG activity. Weak synapses, i.e., elements of the weight vector that are near zero, lie in dimensions that are almost perpendicular to the weight vector, and almost parallel to the hyperplane. By killing and replacing those DG units that have weak synapses to CA3 and mostly perpendicular to *W*, neuronal turnover randomly shifts the patterns in a direction that is mostly parallel to the hyperplane. On average, after this shift, the contexts are easier to separate when synaptic plasticity draws a new weight vector, *W*_*t*+1_, and the cycle continues as such. Though step-to-step improvement on a single instantiation is noisy (Figure 1B, gray trace) the average performance appears to monotonically decrease (Figure 1B, black trace).

To observe the influence of neurogenesis and sparse coding on dimensionality, we observe the singular values, λ(*i*), of the centered DG activity matrix, *S*, corresponding to the standard deviation of activity patterns in the *i*'th dimension. The ratio of λ(*i*)/λ(1) decreases after neurogenesis for all components in both the dense and sparse case, but the decrease is more profound in the sparse case (Figure 4A). To see more clearly how the number of dimensions affects classification performance we observe the effect of restricting the number of components in the weight vector. Observing Equation (28), the weight vector can be decomposed into a sum of weighted components, ${Ŵ}_{d}=\overset{d}{\underset{i}{\sum }}\alpha_{i}u_{i}\;$. We observe how the classification error varies as we incrementally add back components to the weight vector up to dimensionality *d*, plotting a color map of the number of restricted dimensions vs. coding level. Before neurogenesis this map is relatively flat (Figure 4B, left panel), indicating a weak dependence of dimensionality on coding level. In contrast, after neurogenesis the map exhibits a sharp drop in error after a only around 20 components, especially in the sparse coding range around *f* = 0.04 (Figure 4B, right panel). This indicates that neurogenesis reduces the effective dimensionality required for maximal performance at a fixed coding level, and that sparse coding allows for a greater reduction in dimensionality.

### 3.4. The Separation Between Contexts Is Determined by the Context-Bias of Selected DG Neurons

To simplify analysis in the next two sections it is useful to consider training with the matrix of noisy prototype patterns, *S*, rather than the matrix of mean prototype patterns, $\bar{S}$ (See Materials and Methods). We next observe how neuronal competition affects the organization of the DG neuronal population. We define the context-bias of a given DG cell, Ψ_*i*_, as the fraction of patterns it responds to belonging to the (+) context minus those that belong to the (−) context:

(30) $$\begin{array}{l} {\text{Ψ}}_{i}\overset{\text{def}}{=}\left(f_{i}^{+}-f_{i}^{-}\right) \end{array}$$

Therefore, the context-bias takes a value between −1 and +1 and is equal to 0 in cases where a DG cell responds to the same number of (−) and (+) context patterns. For the entire DG population, this can be expressed as the context-bias vector, Ψ,

(31) $$\begin{array}{l} \text{Ψ}=\frac{1}{2P}S\eta^{T}, \end{array}$$

where each column of *S* is a pattern of DG activity, and η is the vector of target CA3 activities (either −1 or +1) for each respective input pattern and is the total number of patterns. The derivation can be found in Equation (15). Note that Ψ is equivalent to the separation between the means of the patterns representing the two respective contexts (See Materials and Methods). Neurogenesis selects for neurons that are biased for each of the two contexts (Figures 5A,B, Top histogram). Therefore, the distribution of Ψ_*i*_ partitions into 3 groups, those that are biased to respond to context (−), those that are biased toward context (+), and newborn randomly generated neurons whose context-bias is centered on zero (Figure 5B). The two biased groups of surviving neurons therefore form an ensemble that can be thought of as memory engrams for their respective contexts. Note that a DG cells context-bias is correlated with its weight to CA3 (Figures 5A,B, scatter plot). On average, the dense case (Figure 5A, top histogram) consists of DG cells that are more biased between the two contexts than the DG cells of the sparse case (Figure 5B, top histogram). This is because the maximum difference between a neurons responsiveness to the two contexts is limited by the total fraction of patterns to which a neuron can respond, i.e., the coding level. With neuronal turnover, in both cases, the average context-bias, and the average CA3 weight increases (Figures 5A,B, top histograms, right histograms, respectively).

We can express the SNR in these terms for a set of training patterns as (See Materials and Methods):

(32) $$\begin{array}{l} \frac{\text{Signal}}{\text{Noise}}=\frac{{\left(W^{T}\text{Ψ}\right)}^{2}}{\sum_{i}W_{i}^{2}f_{i}^{+}\left(1-f_{i}^{-}\right)+\sum_{i}W_{i}^{2}f_{i}^{-}\left(1-f_{i}^{+}\right)-\sum_{i}W_{i}^{2}{\left(f_{i}^{+}-f_{i}^{-}\right)}^{2}} \end{array}$$

The inner product between the DG-CA3 weight vector and the context-bias vector, *W*^*T*^Ψ, determines the SNR between contexts. With neuronal turnover, the increase in absolute weight (Figures 5A,B, side histograms), and absolute context-bias (Figures 5A,B, top histograms) results in increased inner product, *W*^*T*^Ψ, for both the dense and sparse cases (Figure 5G), accounting for the increase in the SNR. However, the SNR grows more quickly in the sparse case (Figure 2G).

### 3.5. Extremely Sparse Coding Allows the Context-Bias of Individual Units to More Closely Determine the Output

We next address the dynamics with which the context-bias and weight vectors change as functions of each other. The purpose of this section is to give mathematical intuition for how neurogenesis takes advantage of sparse coding. In particular, we will discuss how the eigen-components of *W* and Ψ are interacting with each other in the dense coding case and sparse coding case. Note, as described above that the SNR is determined by the product of the weight vector, *W*, and the selectivity vector, Ψ. Furthermore, a DG cells synaptic weight determines its probability of survival. The weight vector is defined as:

(33) $$\begin{array}{l} W^{T}\overset{\text{def}}{=}\eta S^{*}, \end{array}$$

where S^*^ is the pseuodoinverse of the matrix of patterns in DG space. Using the Singular Value Decomposition (see Materials and Methods) we can re-express this in a way that allows us to intuitively understand the relationship between the context-bias vector and the weight vector. First we define ${\hat{\text{Ψ}}}_{i}$ as the projection of the context-bias vector, Ψ, onto the respective *i*-th left singular projection matrix, $u_{i}u_{i}^{T}$.

(34) $$\begin{array}{l} {\hat{\text{Ψ}}}_{i}\overset{\text{def}}{=}u_{i}u_{i}^{T}\text{Ψ} \end{array}$$

As noted above, Ψ is equivalent to the vector of mean separation between the contexts. Therefore, each vector ${\hat{\text{Ψ}}}_{i}$ represents the separation between the context means in the direction of a given singular vector, *u*_*i*_, which expresses the direction of the *i*th largest component of the activity patterns in DG space. ${\hat{\text{Ψ}}}_{i}$ can be thought of as the contribution along the singular vector, *u*_*i*_, to the mean separation between contexts, Ψ. Note that the singular vectors with large singular values represent the most important dimensions of the distribution of patterns in DG space.

Above, we noted that the two contexts separate from each other as neuronal turnover proceeds. Correspondingly, ‖Ψ‖, the euclidean length of Ψ, increases over time (top histograms of Figures 5A,B, and a summary in Figure 5E). However the dense and sparse cases differ in the way dimensionality is reduced. To observe this we now express the weight vector in terms of ${\hat{\text{Ψ}}}_{i}$ as:

(35) $$\begin{array}{l} W^{T}=2P\overset{N}{\underset{i}{\sum }}\sigma_{i}^{-2}{\hat{\text{Ψ}}}_{i}^{T}, \end{array}$$

where 2*P* is a constant scale factor equal to twice the total number of patterns, and σ_*i*_ are the *i*-th singular values of the matrix *S*. The derivation can be found in Appendix B. We see that the weight vector is merely a weighted sum of ${\hat{\text{Ψ}}}_{i}$. Here a tradeoff emerges. Somewhat counterintuitively, the contributions of the ${\hat{\text{Ψ}}}_{i}$ are scaled down by their respective $\sigma_{i}^{-2}$. Thus, though certain singular vectors may represent the mean separation between contexts, their contribution to the weight vector is limited by their singular values. In other words, the more a given ${\hat{\text{Ψ}}}_{i}$ determines the mean separation, the more it is scaled down by its respective $\sigma_{i}^{-2}$.

In order to investigate the difference between dense coding and sparse coding cases, let us look into the distributions of singular values. In Figure 5C, the ranked reciprocals of the square of singular values, $\sigma_{i}^{-2}$, for different cases are presented. The scale-down effect in the dense case is more significant than in the sparse case for the ranks within a neighborhood of the rank 1. Thus, the weight vector in the dense case is subject to more shrinking of the ${\hat{\text{Ψ}}}_{i}$ by their respective $\sigma_{i}^{-2}$ and the elements of the weight vector have a narrower distribution in the dense case than in the sparse case prior to neurogenesis (Figure 5D for individual contributions and side histograms of Figures 5A,B for full distributions).

Due to the differences in scaling factors shown in panel C, ‖*W*‖ has a larger magnitude in the sparse case compared to the dense case despite that ‖Ψ‖ has smaller values, as shown in panel E. In addition to the difference in the magnitude, the normalized inner product (*W*^*T*^Ψ)/(‖*W*‖ ‖Ψ‖) of the sparse case is larger than that of the dense case (Figure 5F), implying that the cosine distance between *W* and Ψ is smaller in the sparse case. In addition, neuronal turnover increases the inner product more rapidly in the sparse case (Figure 5G). Because *W*^*T*^Ψ represents the degree of separation between the presentations of (+) context and (−) context, sparse coding is superior to dense coding in the context separation. This situation is schematically illustrated in Figure 5H.

### 3.6. The Neurogenesis Learning Rule Generalizes to Multiple Contexts

We next analyze patterns of activity in a model with multiple CA3 units to enable the encoding of an arbitrary number of distinct contexts. We use a similar neurogenesis rule in Model 2, in which the DG units compete for trophic signals, except now a DG neurons survival is determined by the sum of the absolute value of its output weights (Figure 6A, see section Materials and Methods) such that those neurons with a sum ranking in the bottom 30% of the population are turned over. In this case we have a weight matrix, *W* in which the elements of each column represents the DG-CA3 weights of a given output CA3 unit. We train the network with 8 contexts and test the network as before, by presenting a novel pattern, μ but now we compare the pattern of CA3 activities represented in the vector ${\hat{\eta }}^{\mu }=\text{sign}\left(W^{T}S\right)$ to the vector representing the target CA3 pattern specified by η^μ^. Requiring a match between these patterns for correct classification, we can then define the error for the μ-th pattern at CA3 as ${\text{err}}^{\mu }=\{\begin{array}{c} 0,\;\text{if}\;{\hat{\eta }}^{\mu }=\eta^{\mu }\\ 1,\;\text{otherwise} \end{array}$. The mean error across test patterns decreases similarly to the generalization error of the two-context case, and again demonstrates the superiority of the sparse case with a coding level of *f* = 0.04 (Figure 6B). One may notice that the less-sparse case with a coding level of *f* = 0.15 has a similar performance level with *f* = 0.04. The setting with *f* = 0.15 may be benefited from the increase in multiplicity in representations for this multiple-context case, *c.f*., Figure 1E. However, the superiority of sparse coding still holds by comparing with the setting with *f* = 0.50.

Similar to the two-context case, the pairwise correlation of the training patterns in DG space demonstrates a clustering after neurogenesis (Figures 6C,D) in which patterns that are members of the same context tend to be correlated. PCA is used to observe the spread of the training patterns in DG space. The training patterns are initially randomly distributed in DG space (Figure 6E) but evolve into separated clusters with neuronal turnover (Figure 6F). To observe the effect of this separation on test patterns that the network has never seen before we project them onto the PCs of the DG representation of the training set, and mark any errors with a gray x (Figures 6G,H). Before neurogenesis, patterns of a given context are often misclassifed due to the lack of separation between the contexts (Figure 6G). After neurogenesis, the separation between training patterns of the contexts (Figure 6F), reduces the probability of such errors on test patterns (Figures 6B,H).

### 3.7. A Model of Synaptic Turnover Achieves Similar Performance With Lower Material Cost

The models analyzed above assume that when a DG neuron has a weak connection to CA3, that neuron dies. However, the turnover rate that yields the best performance is about 30% of DG cells per day for 128 days of neuronal turnover (Figures S1B,C). We therefore explored a model assuming that biology seeks to conserve the material of synapses and neurons that might allow us to predict a realistic rate of neuronal turnover. In Model 3, as in the above models, the connections between DG and CA3 are trained with the pseudoinverse rule. Instead of neuronal turnover of units with weak DG-CA3 weights, we now implement synaptic turnover. A strong connection from the DG to CA3 results in a trophic signal that stabilizes that units EC-DG synapses, while a weak DG-CA3 weight is destabilizing (Figure 7A). We implement stability via the probability of EC-DG synaptic turnover. We assume a linear transfer function (See Materials and Methods, Figure S3A) between a DG units output weight to CA3 and the probability of that units input EC-DG weights being re-randomized, resulting in a random subset of that units EC-DG weights being chosen for re-randomization at each iteration. A slope of 2.5 was optimal in our simulations for the linear transfer function (Figure S3B). This rule results in similar improvement in performance to the prior rule that assumes that a fixed fraction of neurons turnover (Figure 7B). The same geometric intuition as the prior model applies (Figure 7F). The result as before is a reduced dimensionality of the contextual representations, such that reconstruction of an output weight vector that gives maximal cumulative performance (See Materials and Methods) can be achieved with far fewer dimensions (Figure S3C). Similar to Model 1 and 2, the optimal coding level becomes sparser with iterations of turnover (Figures 7C,F).

We then ask, what is the difference among different coding levels in terms of the cellular material turnover required to enable encoding? The total number of synapses turned over in this model is greatly reduced compared to the fixed turnover model, for all coding levels (Figure 7D). Since synaptic stability is thought to determine neuronal survival in several systems (Segal, 2010), including adult-born granule cells in the DG (Doengi et al., 2016), we made a similar assumption in the model to allow us to estimate the rate of neuronal turnover. We chose the conservative assumption that a neuron dies only if all of its synapses are targeted for turnover. With this assumption, the rate of neuronal turnover relative to the previous model drops by two orders of magnitude across all coding levels to range between 0.006 and 0.001 (Figure 7E), similar to the low rate of less than 1% that has been reported in rats (Cameron and McKay, 2001), 0.03–0.06% in the 2 month old mouse (Kempermann et al., 1997b), or 0.004% in humans (Spalding et al., 2013). Our results provide theoretical support to the findings that an extremely low rate of day-to-day neuronal turnover is sufficient to significantly alter memory performance.

The cumulative replacement of preexisting cells with newborn cells is also very low, ranging between 10—22% after 128 days of turnover across all coding levels (Figure 7I) similar to experimental results that have been previously reported in mice (Imayoshi et al., 2008). We see that for the same level of total synaptic or neural replacement, the cumulative error reduction is greater for the sparse case than for the dense case (Figure 7G), implying that sparse coding enables the learning rule to conserve on material turnover.

## 4. Discussion

### 4.1. Neuronal Turnover in a Sparsely Active Dentate Gyrus

It is said to be paradoxical that the DG replenishes its neurons daily even though activity levels are very sparse on average (Piatti et al., 2013). Our results suggest that the sparseness of the DG is actually exploited by adult neurogenesis to find low-dimensional contextual representations that enhance memory encoding (Figures 3C,D). Placing synaptic turnover upstream of neuronal turnover performs similarly (Figure 7), suggesting that similar underlying processes could apply in other systems. As discussed below, such a model may help unify seemingly disparate findings in the neurogenesis literature.

Prior computational models of neurogenesis have implemented neuronal turnover by re-randomization (Chambers et al., 2004; Deisseroth et al., 2004; Becker, 2005; Crick and Miranker, 2006; Chambers and Conroy, 2007; Aimone et al., 2009; Finnegan and Becker, 2015), or by adding new neurons (Weisz and Argibay, 2012) with random synaptic weights. Here we contribute by explicitly addressing the interaction between sparseness and neurogenesis, and evaluating the consequences of a learning rule based on competition for target-derived stability.

The DG is significantly more sparse than most brain regions with a coding level estimated around 0.02–0.04 (Jung and McNaughton, 1993; Leutgeb et al., 2007; Danielson et al., 2016; Diamantaki et al., 2016). In our model, the optimal sparseness for memory encoding evolves to a very sparse coding level as a function of the total amount of time over which the network has undergone encoding via neurogenesis (Figure 1G). This seems to suggest that the sparse code found in the DG may be tuned as such to make the best use of neuronal turnover in memory encoding - though we don't evaluate mechanisms of tuning sparseness, it could be accomplished on a multi-synaptic level such as by feedback inhibition, or by a homeostatic increase in firing threshold.

During neurogenesis, new neurons compete for synaptic contact (Figure 1A). As neurons compete and some replace others, the DG neuronal activities evolve to a low-dimensional representation of the two contexts that are to be learned (Figure 3). In this low-dimensional representation the activity-patterns representing the two contexts are grouped into distinct clusters representing the contexts (Figures 3C,D, 6H).

In a similar framework to ours it was known that there is a limit to how sparse a randomly connected network can be before a tradeoff emerges such that further sparseness actually impairs performance (Barak et al., 2013; Babadi and Sompolinsky, 2014). Babadi and Sompolinsky (2014) demonstrated analytically that the optimality of the sparse code is constrained by amplification of noise by random input weights that is mitigated when a hebbian learning rule is implemented on those weights. Given that hebbian learning structures the input weights to represent correlations among the inputs, they suggested that limitations on the effectiveness of sparse coding might emerge due to the unstructured nature of random weights. We first show that either neuronal (Figure 1B) or synaptic turnover (Figure 7B) improves the performance over the initial condition of random projection studied by Barak et al. (2013) and Babadi and Sompolinsky (2014). Furthermore, we demonstrate that a very sparse code can in fact be optimal even given random input weights (Figures 1G, 7F), implying that fine-tuning, such as the hebbian learning they employed (Babadi and Sompolinsky, 2014), is not always necessary at very sparse coding levels. Instead, via competition for target-derived stability, the sparse code facilitates the search for randomly connected neurons that collectively yield a low dimensional representation of the contextual inputs (Figure 5H).

Decomposing the CA3 weight vector allows us to see the higher correlation between the discriminative components, ${\hat{\text{Ψ}}}_{i}$ and their contribution to the weight vector, $\sigma_{i}^{-2}{\hat{\text{Ψ}}}_{i}$, in the sparse case (Figure 5F). In other words, in the sparse case there exist discriminative components with singular values sufficiently small such that they can be strongly represented in the weight vector.

As a result, with each iteration (day), the synaptic strength of a DG neuron to CA3 can more readily grow in proportion to its contribution to the mean separation between contexts (Figures 5E,F). The overlap between these terms then scales up more quickly in the sparse case (Figures 5F,G). This greater coupling between the mean separation of contexts in the DG and the weights to CA3 (Figure 5D) thereby allows neurogenesis to more rapidly find separated contextual representations in the sparse case (Figures 3C,D). This greater separation allows the network to generalize better to new instances of the same context (Figures 1E, 6B).

### 4.2. Biological Predictions

The major prediction of this study is the dimensionality reduction of contextual codes in the dentate gyrus (DG). This prediction is in principle testable by recording the activity of a population of DG cells that includes both mature and immature neurons during contextual discrimination tasks. Then, analyses similar to those employed in the present study will be applicable to explore how the dimensionality of DG representation evolves during learning and how the dimensionality reduction is affected by the blockade of neurogenesis. Our results are also consistent with several experimental findings. Adult-born neurons are initially hyperexcitable, then gradually acquire the sparse firing characteristics of their mature counterparts (Schmidt-Hieber et al., 2004; Dieni et al., 2013). Correspondingly, input specificity increases with time (Marin-Burgin et al., 2012). This is consistent with the sparsening of the optimal coding level with time in our model (Figures 1G, 7F). Furthermore, if we assume that newborn DG cells initially have very few connections, greater hyperexcitability (higher coding level, f) is necessary for optimal performance (Figures S2B,C).

The preference in our model for an average sparse coding level in the presence of neurogenesis (Figure 1F) is consistent with findings that neurogenesis induces a sparser code in the dentate gyrus (Ikrar et al., 2013) while blockade of neurogenesis results in increased average activity in the dentate gyrus (Burghardt et al., 2012; Lacefield et al., 2012). Meanwhile, increasing the excitability of the DG while neurogenesis is intact may impair contextual discrimination (Jinde et al., 2012).

The initial condition of our model, is equivalent to the encoding of novel contexts. As the contexts become familiar over time, the optimal neurogenesis rate decreases in the neuronal turnover model (Figure S1C), as does the predicted neuronal turnover in the synaptic turnover model (Figure 7E). This is consistent with experimental findings that novelty increases the neurogenesis rate (Kempermann et al., 1997b; Gould et al., 1999). Correspondingly, as the contextual encoding proceeds, their survival rate decreases with time, i.e., exceedingly few adult-born cells survive (Figure S1D). Therefore, relatively few mature cells are replaced and most of the cell death is replacement of immature cells by other immature cells. This is because a very old cell is already part of a favorable representation that enables discrimination and it is improbable to find a new cell that can better contribute. Thus newly adult-born cells have a survival advantage during novel encoding such as would occur during environmental enrichment, similar to what has been found experimentally (Kempermann et al., 1997b; Gould et al., 1999), while mature cells have the advantage under familiarity. Contextual novelty may explain why axonal retraction of mature DG cells results from a losing competition with adult-born cells in the juvenile rat (Yasuda et al., 2011), but not in adult mice in their homecage (Lopez et al., 2012). Since adults have already sufficiently encoded their environment, it is perhaps necessary to expose adults to enriched or novel environments (Kempermann et al., 1997b; Gould et al., 1999) to observe significant outcompeting of mature DG cells by new cells. However, this prediction in survival rate should not be confused with the overall survival rate of all new-born dentate gyrus granule cells. The survival rate mentioned here considers only those dentate gyrus cells able to reach CA3 for competitions. For those newly generated dentate gyrus granule cells failed to emerge into the system, we consider that they are invisible in the model.

Our results are consistent with the presence of high-efficacy, so-called detonator synapses, at the Mossy Fiber (MF) terminals of DG axons to CA3 (McNaughton and Morris, 1987; Jonas et al., 1993; Treves and Rolls, 1994; Henze et al., 1997, 2002; Rollenhagen et al., 2007; Vyleta et al., 2016). The sparse activity of the DG causes the output weights to be larger than in less sparse systems, as the weights of sparse models are of greater magnitude for equivalent context-bias (Figure 5E). Furthermore, neuronal turnover during contextual learning leads to faster growth of the weights in the sparse model compared to those of the dense model (Figure 5E). This is consistent with the experimental finding that contextual learning increases the average synaptic efficacy of MF terminals of axons from the DG to CA3 (Galimberti et al., 2006).

### 4.3. Neuronal vs. Synaptic Turnover

It has been estimated that only around 0.03–0.09% of granule cells are turned over in the adult rodent DG (Kempermann et al., 1997b; Cameron and McKay, 2001), or 0.004% in humans (Spalding et al., 2013). These results have often raised the question - how can such a small number of cells significantly influence behavior (Piatti et al., 2013)? Indeed, there is a stark lack of consensus on whether adult hippocampal neurogenesis always positively correlates with DG-dependent learning (Frankland, 2013; Akers et al., 2014; Lipp and Bonfanti, 2016). Bats show no adult DG neurogenesis for the majority of species studied (Amrein, 2015), though bats clearly exhibit hippocampal place cells, and spatio-contextual reasoning that is attributed to the hippocampus (Finkelstein et al., 2016). Numerous comparative studies have demonstrated heterogeneous adult neurogenesis rates across mammalian species that does not seem to depend on their need for spatial reasoning (Cavegn et al., 2013; Amrein, 2015; van Dijk et al., 2016).

Experimental interventions that suggest a lack of positive correlation between neurogenesis rates and learning of DG-dependent tasks (Wood et al., 2001; Bartolomucci et al., 2002; Holmes et al., 2002; Bizon and Gallagher, 2003; Akirav et al., 2004; Leuner et al., 2004, 2006; Van der Borght et al., 2005), or that learning does not necessarily increase the number of new neurons (van Praag et al., 1999; Döbrössy et al., 2003; Ambrogini et al., 2004; Olariu et al., 2005; Pham et al., 2005; Snyder et al., 2005; Van der Borght et al., 2005), suggest that neuronal turnover is not always the relevant correlate of learning in the DG. Substantial evidence that depletion of neurogenesis does not impair such learning (Shors et al., 2001, 2002; Madsen et al., 2003; Raber et al., 2004; Snyder et al., 2005; Meshi et al., 2006; Frankland, 2013; Groves et al., 2013; Urbach et al., 2013) suggests that molecular mechanisms modulating DG synaptic processes can remain intact and support learning, without requiring neuronal turnover.

Placing synaptic turnover upstream of somatic turnover, as in Model 3 (Figure 7A), may help unify these findings. Synaptic turnover, rather than neuronal turnover may be the relevant measurement with which to correlate DG-dependent learning that is targetable by molecular and cellular interventions in the neurogenic niche. DG neurons compete for CA3 target factors, and those losing the competition have their input synapses destabilized (Figure 7A). If the amount of synaptic destabilization crosses a threshold (in our case, all input synapses destabilized) then the neuron dies. With these assumptions, we indeed find a very low optimal neurogenesis rate (Figure 7E) in the biologically reported range of a fraction of a percent (Kempermann et al., 1997b; Cameron and McKay, 2001; Spalding et al., 2013). This suggests that, via the same form of competition, en masse synaptic turnover could underlie learning, while only a minority of neurons actually turn over. Such a synaptic-turnover-driven neuronal turnover rule is consistent with evidence that activity-dependent competition among mature and immature DG granule cells for CA3 targets (Yasuda et al., 2011), and their input-synaptic stability (Tashiro et al., 2006; Doengi et al., 2016) appears to promote neuronal survival. Furthermore, there is a well-known overlap between factors that influence synaptic plasticity, and those that influence neurogenesis in the DG (Vivar et al., 2013), and many of these same factors influence synaptic stability more generally throughout the central nervous system (Vicario-Abejón et al., 2002). Future behavioral studies in animal models of modulated neurogenesis may benefit from measuring markers of synaptic stability, such as adhesion molecules required for synapse maintenance (Doengi et al., 2016), rather than somatic markers of neurogenesis.

### 4.4. Concluding Remarks

Sparse coding is prevalent throughout many systems of the brain (Barak et al., 2013; Babadi and Sompolinsky, 2014). Our results suggest that neuronal or synaptic turnover in sparsely active regions of the brain may embody a novel learning rule that enhances the clustering of associated activity patterns, and thereby memory encoding and retrieval. Sparseness entails a lower metabolic cost since few neurons are active at any time, and our results further suggest that learning in a sparse layer via turnover conserves synaptic (Figure 7D) or somatic material (Figure 7E), perhaps a previously unrecognized metabolic benefit to sparse coding. The learning curves of all implemented models suggest that differing degrees of sparseness across systems may be found to correspond to the timescale over which they are required to represent memories. Since the optimal sparseness of these models increases as a function of encoding time, we might think of the high sparseness of the DG as being tuned to enable retrieval of episodes that are encoded over long periods of time. Consistent with this timescale, amnesiac patient H.M. lost not only the ability to encode novel information, but also the ability to retrieve memories up to 11 years prior to the removal of his hippocampus (Corkin, 2002). Further investigation of the relationship between synaptic stability and neuronal survival (Doengi et al., 2016) may yield insight into how neuronal turnover and synaptic turnover are coupled. Our work, and that of others (Marin-Burgin et al., 2012; Bergami et al., 2015; Alvarez et al., 2016) suggests that local regulation of sparse activity in the DG may be critical during the addition of new synapses or new neurons that occurs during learning. Similar processes may regulate brain development in general.

## Author Contributions

AD conceived the project and performed mathematical analysis and numerical simulations, and co-wrote the manuscript. CF performed mathematical analysis and numerical simulations, and co-wrote the manuscript. TF supervised the project and co-wrote the manuscript.

### Conflict of Interest Statement

The authors declare that the research was conducted in the absence of any commercial or financial relationships that could be construed as a potential conflict of interest.

## Appendices

### A. Moore Penrose Pseudoinverse Guarantees A Least-square Solution

Let us consider a linear system:

(A1) $$\begin{array}{l} y=Ax\;. \end{array}$$

For a given *y* and a given *A*, we would like to look for a solution to *x* minimizing the square residual given by

(A2) $$\begin{array}{l} \|r{\|}^{2}\equiv \|y-Ax{\|}^{2} \end{array}$$

(A3) $$\begin{array}{l} =y^{T}y-y^{T}Ax-x^{T}A^{T}y+x^{T}A^{T}Ax\;. \end{array}$$

The gradient of the residual is

(A4) $$\begin{array}{l} \nabla_{x}∥r{∥}^{2}=-2A^{T}y+2A^{T}Ax\;. \end{array}$$

$\nabla_{x}∥r{∥}^{2}=0$ implies

(A5) $$\begin{array}{l} A^{T}y=A^{T}Ax\;. \end{array}$$

Suppose *A* is a matrix with linearly independent columns, we have

(A6) $$\begin{array}{l} x={\left(A^{T}A\right)}^{-1}A^{T}y\;. \end{array}$$

Here *A*^*^ ≡ (*A*^*T*^*A*) ^−1^ *A*^*T*^ is the Moore-Penrose pseudoinverse of *A*. The solution to *x* deduced by the Moore-Penrose pseudoinverse should be guaranteed to be the least-square solution.

In our model, the output weights is solved by Moore-Penrose inverse. One may consider replacing η_*T*_ with *y*, ${\bar{S}}^{T}$ with *A* and *W* with *x* in the linear system here. Since $\bar{S}$ is generated from linearly independent input and random input weights, and θ is chosen to match the coding level *f*, the rows of the matrix $\bar{S}$ should also be linearly independent. The argument concerning Moore-Penrose pseudoinverse in this appendix follows.

### Expression of *W* in Terms of $\hat{\text{Ψ}}$

Given that

(A7) $$\begin{array}{l} W^{T}=\eta {\bar{S}}^{*} \end{array}$$

(A8) $$\begin{array}{l} =\eta V\Sigma^{*}U^{T}\;, \end{array}$$

where matrices *U*, *V* and Σ are the matrix given by singular-value decomposition. Note that singular-value decomposition is a generalized eigendecomposition. Diagonal entries of Σ stores eigenvalues of the matrix ${\bar{S}}^{*}$, while matrices *V* and *U* store left-singular and right-singular vectors. On the other hand,

(A9) $$\begin{array}{l} \eta^{T}=2P{\bar{S}}^{*}\text{Ψ} \end{array}$$

(A10) $$\begin{array}{l} \eta =2P{\text{Ψ}}^{T}{\left({\bar{S}}^{*}\right)}^{T}\;. \end{array}$$

Therefore,

(A11) $$\begin{array}{l} W^{T}=\left[2P{\text{Ψ}}^{T}{\left({\bar{S}}^{*}\right)}^{T}\right]V\Sigma^{*}U^{T} \end{array}$$

(A12) $$\begin{array}{l} =2P{\text{Ψ}}^{T}U{\left(\Sigma^{*}\right)}^{2}U^{T}\;. \end{array}$$

Let us consider the entries of *W*^*T*^,

(A13) $$\begin{array}{l} {\left(W^{T}\right)}_{j}=2P\underset{k}{\sum }{\left({\text{Ψ}}^{T}\right)}_{k}\underset{l}{\sum }U_{kl}\underset{m}{\sum }{\left(\Sigma^{-2}\right)}_{lm}{\left(U^{T}\right)}_{mj} \end{array}$$

(A14) $$\begin{array}{l} \;=2P\underset{k}{\sum }{\left({\text{Ψ}}^{T}\right)}_{k}\underset{l}{\sum }{\left(\Sigma^{-2}\right)}_{ll}U_{kl}{\left(U^{T}\right)}_{lj} \end{array}$$

(A15) $$\begin{array}{l} =2P\underset{l}{\sum }{\left(\Sigma^{-2}\right)}_{ll}\underset{k}{\sum }{\left({\text{Ψ}}^{T}\right)}_{k}U_{kl}{\left(U^{T}\right)}_{lj}\;. \end{array}$$

Therefore, the matrix-vector form is given by

(A16) $$\begin{array}{l} W^{T}=2P\underset{i}{\sum }\sigma_{i}^{-2}{\hat{\text{Ψ}}}_{i}, \end{array}$$

where $\hat{\text{Ψ}}\equiv {\text{Ψ}}^{T}u_{i}u_{i}^{T}$ and *u*_*i*_ is the *ith* column of the matrix *U*.
