# Supplementary material for: Hippocampal Neurogenesis Reduces the Dimensionality of Sparsely Coded Representations to Enhance Memory Encoding
Source: Front Comput Neurosci. 2019 Jan 7;12:99. doi: 10.3389/fncom.2018.00099 (PMC6330828; doi:10.3389/fncom.2018.00099)
Supplement: Supplementary file 1 [file Data_Sheet_1.PDF]

Supplementary Material: Hippocampal neurogenesis reduces the dimensionality of sparsely coded representations to enhance memory encoding

1 SUPPLEMENTARY FIGURES

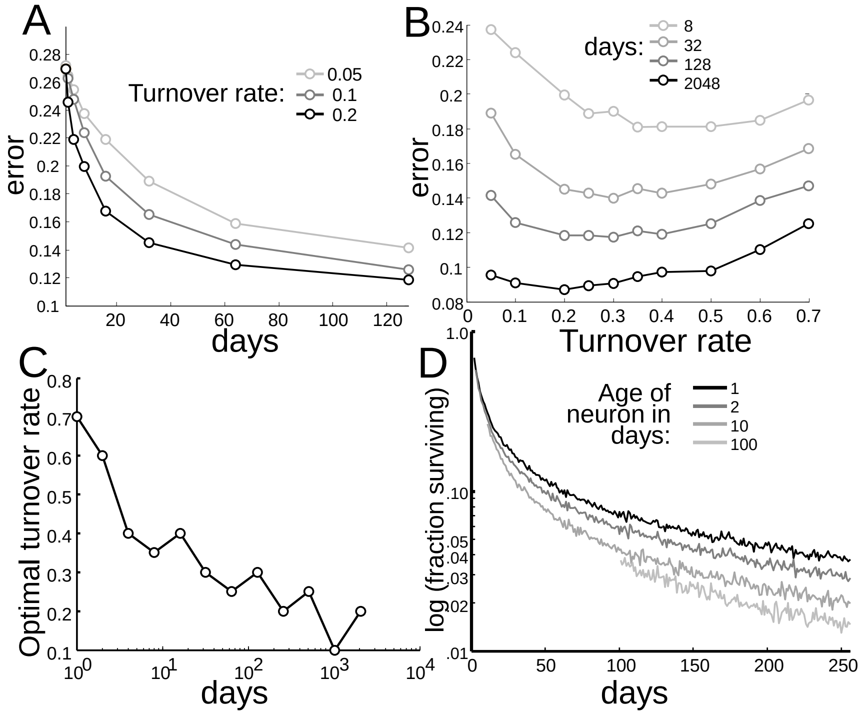

Figure S1: Neuronal Turnover and survival. (A) Generalization error vs number of iterations (days) of turnover for different rates (fraction of neurons turned over per iteration). (B) Generalization error vs turnover rate varying the total number of days of learning. The optimal turnover rate depends upon the total number of iterations of turnover that the network undergoes. (C) Optimal turnover rate decreases with number of iterations of learning. (D) Fraction of surviving neurons as a function of time depending on the age of neuron. Data is for the model with a fixed fraction (0.30) of neurons turned over per day, as in Figures 1 - 5. All results are from the mean of 20 simulations.

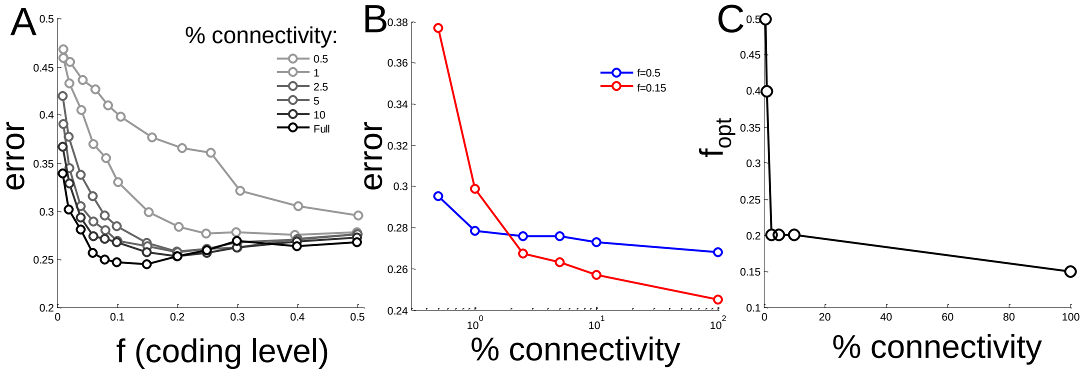

Figure S2: Effect of connectivity on optimal coding level. (A) Generalization error vs coding level, f, varying the percent of neurons in EC to which each neuron in DG receives connections from 0.5% to 100% (Full) connectivity. Fully connected networks lead to lower generalization error (B) Error vs %Connectivity depends upon coding level f. For dense coding of f=0.50 the error curve is relatively flat, but with sparse coding of f=0.15, sparsely connectivity leads to a steep increase in error. (C) Optimal coding level (fopt) vs %Connectivity. Sparse models perform well above some critical level of connectivity that is around 2.5%. All results are the mean of 10 simulations.

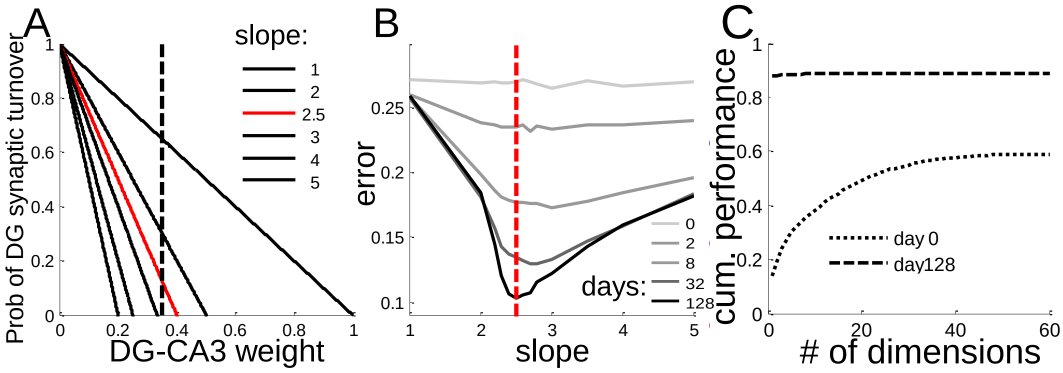

Figure S3: Tuning synaptic turnover. (A) The probability of a EC-DG synapse turning over is proportional to the DG-CA3 output weight of the neuron to which it belongs. (B) The minimum error occurs at a slope of around 2.5, marked by dotted red line, and by solid red line in A, mean of 20 simulations. Therefore a slope of 2.5 was used in the model presented in Figure 7. (C) Performance with reconstructed weight vectors for a 500-DG-neuron x 60-pattern matrix. The rank and dimensionality is 60. Each reconstruction is the linear sum of the indicated number of components (dimensions) times their coefficients determined by the singular value decomposition (See Materials and Methods). After synaptic turnover (day 128) only few components are necessary to achieve full performance indicating a reduction in the dimensionality required to represent the two contexts.
